# Computational modeling of opioid-induced synaptic plasticity in hippocampus

**DOI:** 10.1371/journal.pone.0193410

**Published:** 2018-03-07

**Authors:** Mehdi Borjkhani, Fariba Bahrami, Mahyar Janahmadi

**Affiliations:** 1 CIPCE, Motor Control and Computational Neuroscience Laboratory, School of ECE, College of Engineering, University of Tehran, Tehran, Iran; 2 Neuroscience Research Center and Department of Physiology, School of Medicine, Shahid Beheshti University of Medical Sciences, Tehran, Iran; Western University of Health Sciences, UNITED STATES

## Abstract

According to a broad range of research, opioids consumption can lead to pathological memory formation. Experimental observations suggested that hippocampal glutamatergic synapses play an indispensable role in forming such a pathological memory. It has been suggested that memory formation at the synaptic level is developed through LTP induction. Here, we attempt to computationally indicate how morphine induces pathological LTP at hippocampal CA3-CA1 synapses. Then, based on simulations, we will suggest how one can prevent this type of pathological LTP. To this purpose, a detailed computational model is presented, which consists of one pyramidal neuron and one interneuron both from CA3, one CA1 pyramidal neuron, and one astrocyte. Based on experimental findings morphine affects the hippocampal neurons in three primary ways: 1) disinhibitory mechanism of interneurons in CA3, 2) enhancement of NMDARs current by μ Opioid Receptor (μOR) activation and 3) by attenuation of astrocytic glutamate reuptake ability. By utilizing these effects, simulations were implemented. Our results indicate that morphine can induce LTP by all aforementioned possible mechanisms. Based on our simulation results, attenuation of pathologic LTP achieved mainly by stimulation of astrocytic glutamate transporters, down-regulation of the astrocytic metabotropic glutamate receptors (mGlurs) or by applying NMDAR’s antagonist. Based on our observations, we suggest that astrocyte has a dominant role in forming addiction-related memories. This finding may help researchers in exploring drug actions for preventing relapse.

## Introduction

Addiction is a chronic brain disease, in which addicted patients continually exhibit drug-seeking behavior despite its adverse consequences. In an addicted person, cellular and molecular adaptations in particular brain regions lead to behavioral abnormalities [1, 2]. Drugs induce plasticity in the functions of neurons and synapses. These modifications can be seen as a form of "molecular or cellular memory" [3]. In the addiction process, abnormal memories are stored in specific regions of the brain, including the hippocampus, amygdala, prefrontal cortex, Ventral Tegmental Area, Nucleus Accumbens [2, 3]. As a matter of fact, these abnormal memories are responsible for addiction syndromes. Drug-induced changes in the functions of glutamatergic synapses have been considered to be a significant cause of drug-associated memories [2–6]. Contextual information of drug consumption is stored in the hippocampus and plays a vital role in drug-related reward property [6, 7] and the relapse [8].

Opioids that are used for the treatment of pain are intensively addictive. Opioids similar to other drugs may have long-term effects on the affected neurons through Long-Term Potentiation (LTP) or Long-Term Depression (LTD) mechanisms which are thought to be the cellular basis of memory formation [1, 2]. Many opioids, including morphine, affect the neurons through μ opioid receptors, which are G-protein coupled receptors (GPCRs). In the presence of morphine, μOR activation can lead to induction of LTP/LTD [9]. Therefore, assessing opioid-induced LTP/LTD could be the key to understanding the mechanism by which opioids induce addiction [2, 10]. Some animal research indicates that morphine affects the hippocampal synapses by inducing LTP in the injected areas, and thereby may trigger the addiction process [11, 12].

Opioid-induced induction of LTP in hippocampal neurons is via a disinhibitory mechanism which affects the hippocampal interneurons [13–17]. Opioids cause an inhibition of GABA release from hippocampal interneurons [16, 17]. So, pyramidal neurons of CA3 receive less GABA, and inhibitory postsynaptic potentials (IPSPs) in CA3 neurons attenuate [18]. It seems that inhibition of interneurons by opioids is due to the enhancement of *K*^+^ channel activity, or due to the reduction of *Ca*^2+^ channel currents [19]. It has also been shown that inhibition of *Ca*^2+^ channels by morphine requires G-proteins activation [20, 21].

On the other side, astrocyte has a dominant role in the induction of LTP and synaptic plasticity [22–27]. Furthermore, astrocyte involves in opioid dependency [28, 29]. It has been shown that the main player of astrocyte’s contribution to opioids dependency is its important role in homeostasis, which mainly mediates by astrocytic transporters [28–30].

The goal of this paper is to present a biophysically-induced mathematical model at the synaptic level that can denote some features of the cellular addiction formation by opioids in hippocampus. Based on the experimental evidence, we analyze the effects of opioids on LTP at the synaptic level due to the activation of μORs using the proposed mathematical model.

In the first part of the paper, neurobiology of opioid signaling in synaptic plasticity and memory formation will be described. The second part introduces the modeling approach to describe the opioid signaling process at the synaptic level for the induction of pathological LTP. In the third part, simulation results are shown for normal and pathological conditions. Finally, discussion and conclusion of the results are presented.

## Neurobiology of opioid signaling in synaptic plasticity and addiction memory formation

**Fig 1:**
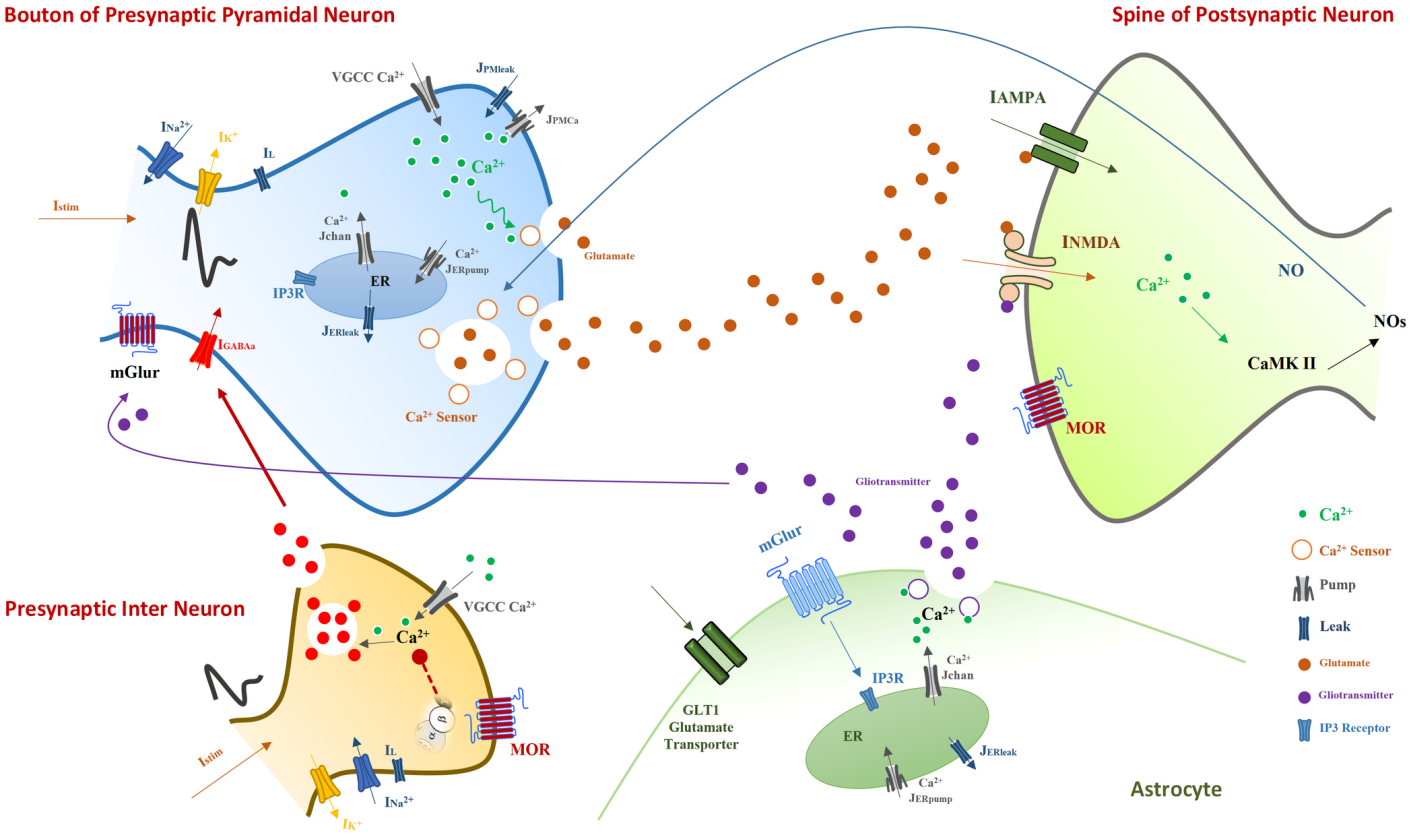
Neurobiology of opioid signaling studied in this work. The model consists of a pyramidal neuron’s bouton and a GABAergic single compartment interneuron from CA3 layer, and a spine of a postsynaptic CA1 pyramidal neuron and an astrocyte. The astrocyte monitors the synaptic transmission, regulates presynaptic glutamate release and is involved in the postsynaptic transmission by releasing gliotransmitters.

It has been shown that glutamatergic synapses play an indispensable role in the formation of pathological addiction related memories [5, 31–33]. Furthermore, LTP/LTD induction is supposed to be a key player in synaptic plasticity and memory formation. Here, we attempt to describe how morphine can modulate the LTP induction in the CA3-CA1 region of the hippocampus and how it may contribute to the pathological memory formation (Fig 1).

Morphine activates μORs of hippocampal interneurons [18]. Replacement of Guanosine Diphosphate to Guanosine Triphosphate occurs in morphine-sensitive GPCRs. In the following, the *α* subunit is disconnected from *βγ* dimer. Separated subunits are capable of modulating *Ca*^2+^ channels. For example, *Gα* modulates calcium channels in a slow and indirect way through second messengers. On the other side, *Gβγ* directly affects *Ca*^2+^ channels which cause them to go to a reluctant state [34]. Thus, morphine seems to inhibit the voltage-gated *Ca*^2+^ channels of interneurons [19]. Inhibition of *Ca*^2+^ channels in turn leads to less *Ca*^2+^ influx into the interneurons. The consequence of less *Ca*^2+^ entry is the suppression of GABA release from interneurons. Thus, GABAergic receptors of CA3 pyramidal neurons receive less GABA, which causes more neuronal excitation [18]. Further excitation of pyramidal neurons leads to more glutamate release, which means CA1 pyramidal neurons and astrocytes are going to be more excited than normal level. Furthermore, opioids decrease astrocytic glutamate transporter1 (GLT1) activation which is responsible for the glutamate uptake from the synaptic cleft [28, 30]. As a result, reuptake of glutamate is reduced by astrocyte, and therefore more glutamate can be found in the synaptic cleft. Enhancement of glutamate in synaptic cleft conveys to more excitation of the postsynaptic CA1 neurons.

Opioids also affect the CA1 postsynaptic pyramidal neurons. In fact, it removes the blocking effect of *Mg*^2+^ on N-methyl-D-aspartate (NMDA) receptors and thereby increases the fast glutamatergic transmission [14, 31, 35]. The outcome of this process is the augmentation of calcium entry into the postsynaptic neurons [36, 37]. Enhancement of calcium concentration in the postsynaptic neurons results in phosphorylation of α-amino-3-hydroxy-5-methyl-4-isoxazolepropionic acid (AMPA) receptors and Nitric Oxide (NO) synthesis by *Ca*^2+^ Calmodulin-dependent protein kinase II (CAMKII) mechanisms [31, 38]. Then, NO retrogradely diffuses to the presynaptic CA3 pyramidal neuron and increases the binding capability of presynaptic calcium sensors, which may cause more glutamate release and induction of LTP in the synapse [31, 39, 40].

## Modeling approach

**Fig 2:**
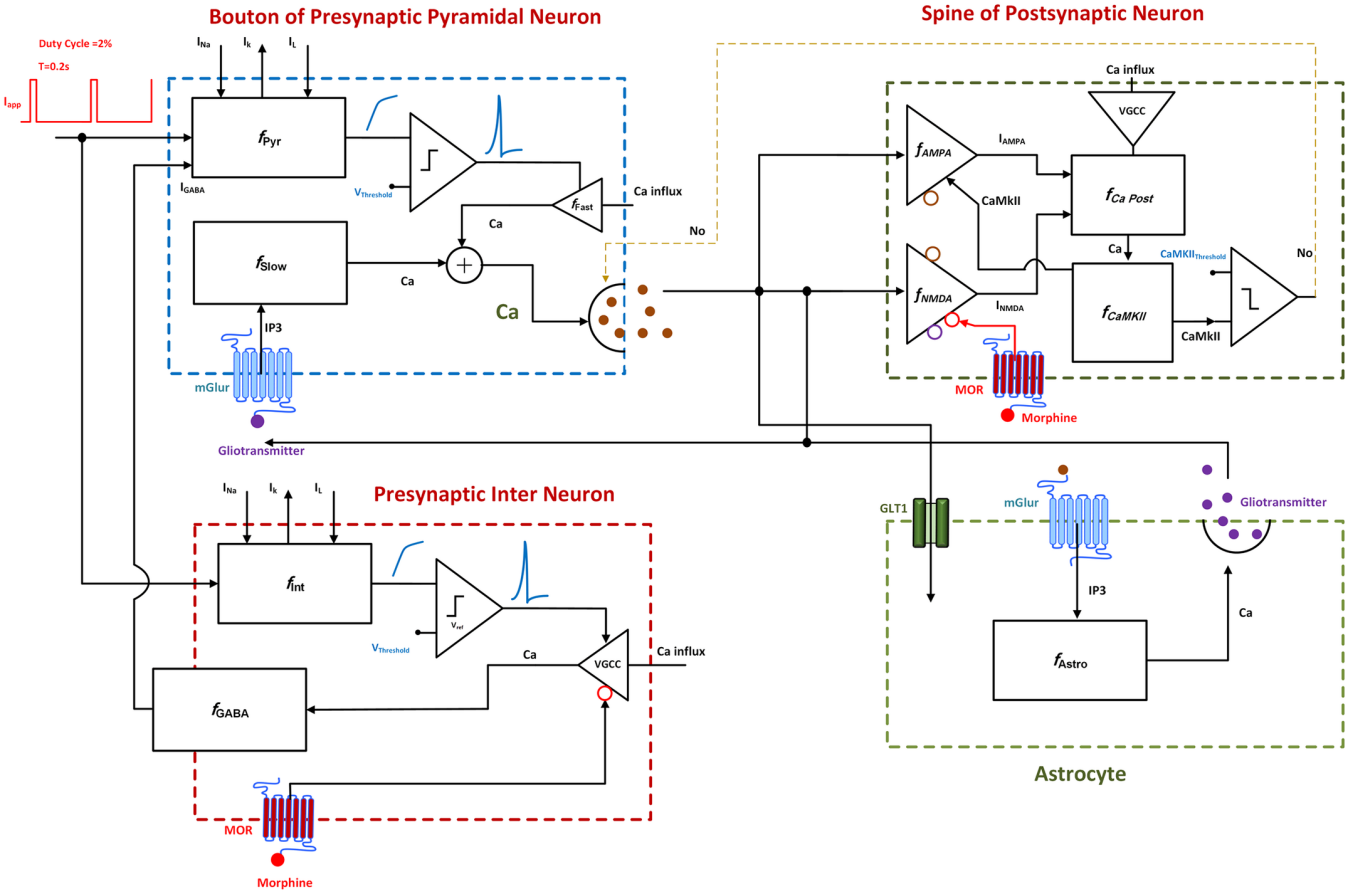
Block diagram of different parts of the proposed model. External stimulation is followed by action potential generation in the presynaptic pyramidal neuron and the interneuron. Consequently, level of presynaptic calcium is elevated through two fast and slow mechanisms. Fast calcium oscillations are due to APs, and slow ones are because of IP3 production. Gliotransmitters activate mGlurs and contribute to slow calcium oscillations by IP3 production. Calcium enhancement results in glutamate release. Interneuron’s activation modulates GABA receptors in pyramidal neuron and is sensitive to the opioid receptor’s activation. Released glutamate reaches to the postsynaptic neuron and the astrocyte. The astrocyte releases gliotransmitter which affects presynaptic and postsynaptic neurons. Functions of AMPARs and NMDARs are considered in the postsynaptic neuron. Activation of the receptors enables CaMKII phosphorylation process. Exceeding phosphorylated CaMKII from a threshold leads to NO production which retrogrades to the presynaptic neuron. Finally, postsynaptic opioid receptors modulate NMDARs activation.

As mentioned earlier, the goal of this paper is to present a biophysically-driven mathematical model at the synaptic level in hippocampus in order to describe the cellular addiction formation by opioids. The main components of the proposed model are: (1) a bouton of a presynaptic CA3 pyramidal neuron, which can produce action potentials (APs) by receiving external stimulations and generates NO which can act as a retrograde signal that enhances the release of glutamate, (2) a GABAergic single compartment interneuron at the CA3 layer which inhibits the CA3 pyramidal neuron, (3) a spine of a postsynaptic CA1 pyramidal neuron which can produce Excitatory Postsynaptic Potentials (EPSPs) based on the amount of glutamate in the synaptic cleft; functions of NMDA and AMPA receptors of the postsynaptic neuron are also considered in this model, and (4) an astrocyte which monitors the synaptic transmission, regulates presynaptic glutamate release and is involved in the postsynaptic transmission by releasing gliotransmitters. The block diagram of different parts of the model is presented in Fig 2.

### Presynaptic neurons

For modeling the presynaptic CA3 pyramidal cell and interneuron, we have utilized Hodgkin-Huxley (H-H) type formulation to generate APs in presynaptic neurons [41]:

$$\frac{dV_{pre}}{dt}=f_{Pyr}(I_{app},{\text{I}}_{Na},{\text{I}}_{K},{\text{I}}_{L},{\text{I}}_{GABA_{A}})$$ (1)

$$C\frac{dV_{Int}}{dt}=f_{Int}(I_{app},I_{Na},I_{K},{\text{I}}_{L})$$ (2)

APs can be generated due to externally applied current (*I*_*app*_) in the presence of sodium (*I*_*Na*_), potassium (*I*_*K*_) and leak (*I*_*L*_) ionic currents (full description of *f*_*Pyr*_ and *f*_*Int*_ can be found in Appendix). Besides, we have adapted and added a GABAergic inhibitory current ($I_{GABA_{A}}$) to the presynaptic pyramidal neuron similar to the formulation used by Zachariou *et al*. [42]:

$$I_{GABA_{A}}=10g({\text{V}}_{pre}+80)$$ (3)

In Eq (3), *g* is the ratio of activated *GABA*_*A*_ receptors and is described by [34, 42]:

$$\frac{dg}{dt}=\frac{g_{\infty }({\text{V}}_{Int})-\text{g}}{\tau_{g}},\;g_{\infty }({\text{V}}_{Int})=\frac{1}{1+e^{-({\text{V}}_{Int}-100(1-CaCh))/5}}$$ (4)

Here, *g*_∞_(V_*Int*_) is assumed to be a sigmoid function which its half maximum voltage depends on activation of the interneuron calcium channels (*CaCh*). *τ*_*g*_ = 1 ms represents time constant of *GABA*_*A*_ receptor.

Hippocampal opioid receptors which are activated by morphine are mostly μORs. These receptors belong to GPCRs which are activated by morphine or its agonists. Because of opioids disinhibitory effect on the synaptic transmission in the hippocampus, it is supposed that presynaptic opioid receptors are on the hippocampal interneurons. Recently, Zachariou and colleagues proposed a model for hippocampal cannabinoid receptors (CBRs) [42]. This model is based on Bertram’s minimal model of G-protein activation [34]. Since μORs and CBRs are both GPCRs, we have adapted this model to describe the activation of μORs in the hippocampus. The presence of morphine (*Morph*) results in activation of μORs (*MOR*) which modulates calcium channels activation (*CaCh*). Equations derived from [34, 42] are modified as follows for opioid receptors:

$$\frac{d(MOR)}{dt}=\frac{MOR_{}^{\infty }-MOR}{1000}$$ (5)

MOR∞=hMOR(Morph)=21+(0.1Morph)1.2 (6)

$$\frac{d(\text{CaCh})}{dt}=k_{-}(1-CaCh)-{\text{k}}_{+}CaCh$$ (7)

$$k_{-}=\frac{0.3}{1+e^{\frac{-V_{Int}}{5}}},{\text{k}}_{+}=0.0006MOR$$ (8)

Maximum number of activated μORs is described by *MOR*^∞^. This parameter depends on morphine concentration (*Morph*). *k*_ Shows reluctant to willing transition rate of calcium channel and depends on interneuron’s voltage (*V*_*Int*_), this parameter reflects the voltage-dependent dissociation of *G*_*βγ*_ from the channel, k_+_ is the transition rate of the willing to reluctant state for calcium channel and depends on morphine concentration, this parameter reflects the concentration of the activated G-proteins. It can be seen that morphine presence activates μORs which is described in Eqs (5) and (6). Consequently, calcium channels activation in interneuron changes in Eqs (7) and (8). Therefore, the conductance for inhibitory current varies in Eq (3) due to changes in Eq (4). Here, for the sake of simplicity, like Bertram *et al*., we assumed no dynamics for the neurotransmitter release from presynaptic interneuron to the pyramidal postsynaptic neuron [34]. Instead of that, we assumed that activation of VGCCs in interneuron affects directly the conductance of *GABA*_*A*_ receptors in the presynaptic pyramidal neuron [34].

After APs are generated in presynaptic pyramidal neuron, glutamate will be released. Glutamate release depends on calcium concentration in the neuron. Calcium concentration in the presynaptic neuron can change through fast (*c*_*Fast*_) and slow (*c*_*Slow*_) dynamics which is described by Eq (9).

$$c_{i}^{}=c_{Fast}+c_{Slow}$$ (9)

Fast calcium oscillations which occur due to Aps, are governed by:

$$\frac{dc_{Fast}}{dt}=f_{Fast}({\text{I}}_{Ca},{\text{I}}_{PMCa},{\text{I}}_{PMleak})$$ (10)

Here, *I*_*Ca*_ represents calcium current through N-type channels, *I*_*PMCa*_ denotes calcium current due to ATPase pump which pumps extra calcium out of the cell, and *I*_*PMleak*_ is the leak current from extracellular space into Bouton (Details about *f*_*Fast*_ can be found in the Appendix).

Slow calcium oscillations occur due to the activation of the extrasynaptic mGlurs. Since mGlurs activation has an indispensable role in synaptic plasticity governed by opioids, we have considered its role in the model. Activation of mGlurs is due to the binding of gliotransmitters. The presence of gliotransmitters lead to Inositol trisphosphate (IP3) production in an intracellular space which enhances calcium concentration in the cell using endoplasmic reticulum (ER) calcium resources. This process is modeled using modified Li-Rinzel model introduced by Tewari and Majumdar [22, 43]:

$$\frac{dc_{Slow}}{dt}=f_{Slow}({\text{J}}_{chan},{\text{J}}_{ERpump},{\text{J}}_{ERleak})$$ (11)

Here *J*_*chan*_ denotes calcium influx from ER into cytosol through the IP3 receptor, *J*_*ERpump*_ is the pumped calcium from intracellular space to ER and *J*_*ERleak*_ indicates leaked calcium ions from ER into intracellular space (Details about *f*_*Slow*_ can be found in the Appendix).

Presynaptic *Ca*^2+^ ions can bind to *Ca*^2+^ sensors of glutamate vesicles and contribute in vesicle release in the synaptic cleft [22, 23, 44]. The kinetic model for the calcium binding to the calcium sensor is governed by:

$$X\overset{5\alpha c_{i}}{\underset{\beta }{⇄}}X{\left({\text{c}}_{i}\right)}_{1}\overset{4\alpha c_{i}}{\underset{2\beta }{⇄}}X{\left({\text{c}}_{i}\right)}_{2}\overset{3\alpha c_{i}}{\underset{3\beta }{⇄}}X{\left({\text{c}}_{i}\right)}_{3}\overset{2\alpha c_{i}}{\underset{4\beta }{⇄}}X{\left({\text{c}}_{i}\right)}_{4}\overset{\alpha c_{i}}{\underset{5\beta }{⇄}}X{\left({\text{c}}_{i}\right)}_{5}\overset{\gamma }{\underset{\delta }{⇄}}X{\left({\text{c}}_{i}\right)}_{5}^{*}$$ (12)

Here, α is the rate of *Ca*^2+^ association constant and β is the rate of *Ca*^2+^ dissociation constant. γ, δ and β are constants which describe calcium-independent isomerization. X shows the *Ca*^2+^ sensors on vesicles without any *Ca*^2+^ bound. *X*(*c*_*i*_)_1_ denotes sensor with one *Ca*^2+^ bound, *X*(*c*_*i*_)_5_ describes sensor with five *Ca*^2+^ bound and finally $X{\left(c_{i}\right)}_{5}^{*}$ is the isomer of *X*(*c*_*i*_)_5_ which is ready to release.

In addition to evoked release due to the APs, the release of vesicles can occur spontaneously when there is no depolarization in the membrane. This type of release also depends on presynaptic *Ca*^2+^ concentration and can occur when the *Ca*^2+^ concentration is low in a non-depolarized membrane [44, 45]. Spontaneous release rate due to *Ca*^2+^ concentration is modeled by Nadkarni and Jung [45] using Poisson process and is expressed as follows:

$$\lambda \left(C_{i}\right)=a_{3}{\left(1+\text{exp}\left(\frac{a_{1}-c_{i}}{a_{2}}\right)\right)}^{-1}$$ (13)

Where, *a*_1_ = 50*μM*, *a*_2_ = 5*μM* and *a*_3_ = 0.85*ms*^−1^ are the *Ca*^2+^ concentration at which *λ* is halved, slope factor of spontaneous release rate *λ*, and maximum spontaneous release rate, respectively. Values for these parameters are determined by Tewari and Majumdar such that, the frequency of spontaneous release is in the range of 1–3 Hz. Experimental findings indicate that this is a valid frequency range in the presence of astrocyte [22]. In general, glutamate release dynamics are depicted as follows [22]:

$$\frac{dR}{dt}=\frac{I}{\tau_{rec}}-f_{r}R,\frac{dE}{dt}=-\frac{E}{\tau_{inac}}+f_{r}R,I=1-R-E$$ (14)

Where, the fraction of ready to release vesicles is denoted by *R*, effective vesicles in synaptic cleft is represented by *E*, and inactive vesicles rate is denoted by *I*. *τ*_*inac*_ = 3*ms* is the time constant of vesicle inactivation and *τ*_*rec*_ = 800*ms* is the time constant of vesicle recovery. *f*_*r*_ is the probability of vesicle release and has values (0, 0.5, 1). The total number of vesicles that can be released is assumed to be two vesicles. When two vesicles get ready to be released, the value of *f*_*r*_ is 1, when one vesicle is ready to release value of *f*_*r*_ is 0.5, and finally, when there are not any vesicles to be released *f*_*r*_ is 0. The number of vesicles that get ready to be released can be calculated by stochastic simulation of Bollmann kinetic model for evoked release (by APs) [44] or by using Poisson function which refers to spontaneous release [22, 23]. There is also an inactivation time of 6.34 ms for vesicle release (evoked or spontaneous release) [45].

Glutamate concentration in synaptic cleft can be altered due to some factors which are modeled by Tewari and Majumdar in the CA3-CA1 synapse. The fraction of effective vesicles, glutamate concentration in vesicles and docked vesicles and also degradation rate of glutamate in synaptic cleft can be considered as significant factors. Glutamate concentration can be represented by [22] Eq (15).

$$\frac{dg}{dt}=n_{v}.g_{v}.E-g_{c}.g$$ (15)

Here, *g* is the concentration of glutamate in the synaptic cleft, *n*_*v*_ = 2 and *g*_*v*_ = 60*μM* are the number of docked vesicles and glutamate concentration in vesicles respectively. *g*_*c*_ = 10*ms*^−1^ is the degradation rate of glutamate which relates to neurotransmitter reuptake by astrocyte. Neurotransmitter reuptake is one of the major factors in the processing of addiction associated LTP formation and would be analyzed in this paper.

### Astrocyte

Astrocytes release gliotransmitter, which can affect the synaptic transmission. Gliotransmitter release by astrocyte depends on the *Ca*^2+^ concentration in astrocyte. The Released gliotransmitter can influence the presynaptic neuron through mGlurs, then postsynaptic neuron through NMDARs. In the proposed model, vesicle release dynamics by astrocyte are based on the Tewari and Majumdar’s model [22]. Furthermore, It has been shown that during consumption of opioids, the astrocytic ability for reuptake is attenuated [28, 29]. A decrease in astrocyte’s glutamate reuptake ability is a primary cause for LTP induction that has been assessed during our simulation studies.

Released glutamate in the synapse, activates mGlurs of astrocyte, which leads to the augmentation of astrocytic *Ca*^2+^ concentration. *Ca*^2+^ Changes in astrocyte are because of *IP*_3_ mechanisms. *Ca*^2+^ oscillations in astrocyte is described by [46]:

$$\frac{dc_{Astro}}{dt}=f_{Astro}(J_{chan,a},J_{pump,a},J_{leak,a})$$ (16)

Where *c*_*Astro*_ denotes *Ca*^2+^ concentration in intracellular space of astrocyte, *f*_*Astro*_ denotes the function which relates calcium currents to the calcium concentration. *J*_*chan*,*a*_ is *Ca*^2+^ flux from ER to the intracellular space due to *IP*_3_ binding to ER’s *IP*_3_*R*, *J*_*pump*,*a*_ is the *Ca*^2+^ removing from intracellular space by SERCA pump, *J*_*leak*,*a*_ is the *Ca*^2+^ leak from ER to intracellular space (detailed information about *f*_*Astro*_ can be found in the Appendix).

Equations which describe vesicle release dynamics by astrocyte are [22] given by Eq (17).

$$\begin{array}{l} \frac{dR_{a}}{dt}=\frac{I_{a}}{\tau_{rec}^{a}}-\Theta \left(c_{Astro}-c_{Astro}^{thresh}\right).{\text{Pr}}_{a}.R_{a},\frac{dE_{a}}{dt}=-\frac{E_{a}}{\tau_{inact}^{a}}+\Theta \left(c_{Astro}-c_{Astro}^{thresh}\right).Pr_{a}.R_{a},\\ I_{a}=1-R_{a}-E_{a} \end{array}$$ (17)

Here *R*_*a*_ denotes releasable vesicles in astrocyte, *E*_*a*_ shows effective vesicles, *I*_*a*_ is the inactive vesicles. Θ is Heaviside function, $c_{Astro}^{thresh}=196.69nM$ is the threshold for calcium that can contribute to vesicle release, $\tau_{inact}^{a}=3ms$ denotes to inactivation time constant and $\tau_{rec}^{a}=800ms$ shows recovery time constant.

It is suggested that three independent gates are responsible for gliotransmitter release and each of the gates need a calcium ion to bind [22, 47]. So, at least three calcium ions are needed for opening of the gates to permit vesicle release from astrocyte. This is described by [47] and depicted as Eq (18).

$$\frac{dO_{j}}{dt}=k_{j}^{+}.c_{Astro}-\left(k_{j}^{+}.c_{Astro}+k_{j}^{-}\right).O_{j}$$ (18)

Here *O*_*j*_ denotes the opening probability of the gate, $k_{\text{j}}^{+}$ is the opening rate of the gate, $k_{\text{j}}^{-}$ is the closing rate of the gate and *c*_*Astro*_ denotes calcium concentration ($k_{1}^{+}=3.75*10^{-3}/\mu M.ms$,$k_{1}^{-}=4*10^{-4}/ms$,$k_{2}^{+}=2.5*10^{-3}/\mu M.ms$,$k_{2}^{-}=1*10^{-3}/ms$,$k_{3}^{+}=1.25*10^{-2}/\mu M.ms$,$k_{3}^{-}=1*10^{-3}/ms$). So Pr_*a*_ = *O*_1_.*O*_2_.*O*_3_ denotes opening probability of a vesicle release site. Finally, Gliotransmitter dynamics in synapse is described by [22]:

$$\frac{dg_{a}}{dt}=n_{a}^{v}.g_{a}^{v}.E_{a}-g_{a}^{c}.g_{a}$$ (19)

Here *g*_*a*_ indicates gliotransmitter concentration, $n_{a}^{v}=12$ denotes the number of vesicles, $g_{a}^{v}=20mM$ represents gliotransmitter concentration in one vesicle and $g_{a}^{c}=10{\left(ms\right)}^{-1}$ shows clearance rate of glutamate.

### Postsynaptic neuron

Spine of the postsynaptic neuron is a passive membrane which can produce EPSPs. Model of the spine consists of AMPA and NMDA receptors. Binding synaptic glutamate to AMPARs depolarizes the membrane potential. A depolarized membrane, potentially activates NMDARs in the presence of neurotransmitter and gliotransmitter. Activation of NMDARs allows the *Ca*^2+^ ions to flow to the neuron. *Ca*^2+^ entry precedes to CaMKII phosphorylation and NO synthesis which can retrograde to the presynaptic neuron. Phosphorylated CaMKII also lead to AMPAR insertion on the cell membrane, which is one of the signs of memory formation in synaptic scale. In the presence of morphine, NMDAR’s current enhances because of morphine’s modulation on channel conductance and morphine-induced inhibition of *Mg*^2+^ blockage on NMDARs activity [14, 31, 35]. This enhancement of NMDAR current by morphine is dose dependent and was considered in the model.

The CA1 pyramidal neuron’s spine modeled using equations [48]:

$$\tau_{post}\frac{dV_{post}}{dt}=-({\text{V}}_{post}-{\text{V}}_{post}^{rest})+{\text{R}}_{m}I_{syn}$$ (20)

Here *τ*_*post*_ denotes time constant of neuron membrane, ${\text{V}}_{post}^{rest}$ shows neuron’s membrane potential at rest, R_*m*_ is the actual resistance of spin, *I*_*syn*_ is synaptic current and can be described by:

$$I_{syn}=-({\text{I}}_{AMPA}+{\text{I}}_{NMDA})$$ (21)

Where I_*AMPA*_ and I_*NMDA*_ are AMPA and NMDA receptors mediated currents respectively. AMPAR current considered using Destexhe’s model [49]:

$$I_{AMPA}=f_{AMPA}(g_{AMPA},m_{AMPA},{\text{V}}_{post})$$ (22)

Where V_*AMPA*_ is reversal potential of the receptor, V_*post*_ denotes membrane potential, *m*_*AMPA*_ represents gating variable of AMPAR. *g*_*AMPA*_ is the conductance of AMPAR and changes due to CaMKII phosphorylation process:

$$g_{AMPA}=f_{g_{AMPA}}(\text{CaMKII})$$ (23)

Further information about AMPAR phosphorylation can be found in Castellani et all’s papers [50, 51].

Furthermore, in the proposed model, *I*_*NMDA*_ modeled using Moradi et all’s formulation with a slight modification:

$$I_{NMDA}=f_{NMDA}(g_{NMDA},m_{NMDA},Mg,{\text{V}}_{post})$$ (24)

*f*_*NMDA*_ represents a function that relates *I*_*NMDA*_ to the receptor conductance (*g*_*NMDA*_), gating variables of receptor (*m*_*NMDA*_), *Mg*^2+^ blocking (*Mg*) and postsynaptic membrane potential (V_*post*_). Here in the proposed model, *Mg*^2+^ blocking depends on morphine concentration (Morph) and membrane potential (*V*_*post*_) through *f*_*Mg*_:

$$Mg=f_{Mg}(\text{Morph},{\text{V}}_{post})$$ (25)

Moreover, NMDAR gating variable is governed by:

$$\frac{dm_{NMDA}}{dt}=f_{m_{NMDA}}(\alpha_{NMDA},\beta_{NMDA},g_{pre},g_{Astro})$$ (26)

Here V_*NMDA*_ is the reversal potential of NMDAR, *α*_*NMDA*_ and *β*_*NMDA*_ are opening and closing rate of receptor respectively, *g*_*NMDA*_ is NMDA channels conductance which is described by:

$$g_{NMDA}=f_{g_{NMDA}}(\text{Morph},g_{VD})$$ (27)

Which depends on the *g*_*VD*_ (voltage dependent conductance) and morphine concentration through $f_{g_{NMDA}}$.

Enhancement of postsynaptic calcium is through AMPARs, NMDARs, and voltage-gated calcium channels and it descends is through calcium pumps. Postsynaptic calcium concentration can be described by [22]:

$$\frac{dc_{post}}{dt}=f_{Ca_{Post}}({\text{I}}_{AMPA},{\text{I}}_{NMDA},{\text{I}}_{CaL},{\text{S}}_{Pump})$$ (28)

Here, *c*_*post*_ denotes postsynaptic calcium concentration which depends on AMPAR current, and is denoted by *I*_*AMPA*_, NMDA current is showed by *I*_*NMDA*_, voltage-gated calcium channels activation is described by *I*_*CaL*_ and pumped calcium is defined by *S*_*pump*_.

Postsynaptic *Ca*^2+^ concentration variation may lead to CaMKII phosphorylation which is governed by equations [52]:

$$Ph.CaMKII=f_{CaMKII}({\text{c}}_{Post})$$ (29)

Finally enhancement of presynaptic calcium sensors sensitivity due to NOs is governed by a sigmoidal function:

$$\frac{K_{syt}}{1+e^{\left(-\frac{\left((\sum_{1}^{10}P_{i})-P_{1/2}\right)}{k_{1/2}}\right)}}$$ (30)

Here, $P_{\frac{1}{2}}=40\;\mu M$ is the half of total CamKII, *k*_*syt*_ = 0.5% and *k*_1/2_ = 0.4 *μM* are constants. Sensitivity of calcium sensors in Eq (12) enhances through this sigmoidal function.

## Results

Simulation results of the proposed model are presented in this section. Simulations were implemented in Matlab 2014a software. Forward Euler method with fixed step size of 0.05 msec was used to solve the differential equations. Presynaptic neurons stimulated with a pulse train with 10 *μA*/*cm*^2^ density (5 Hz with a duration of 4ms). Moreover, simulation time is 60 sec.

### Mu-opioid receptor activation

**Fig 3:**
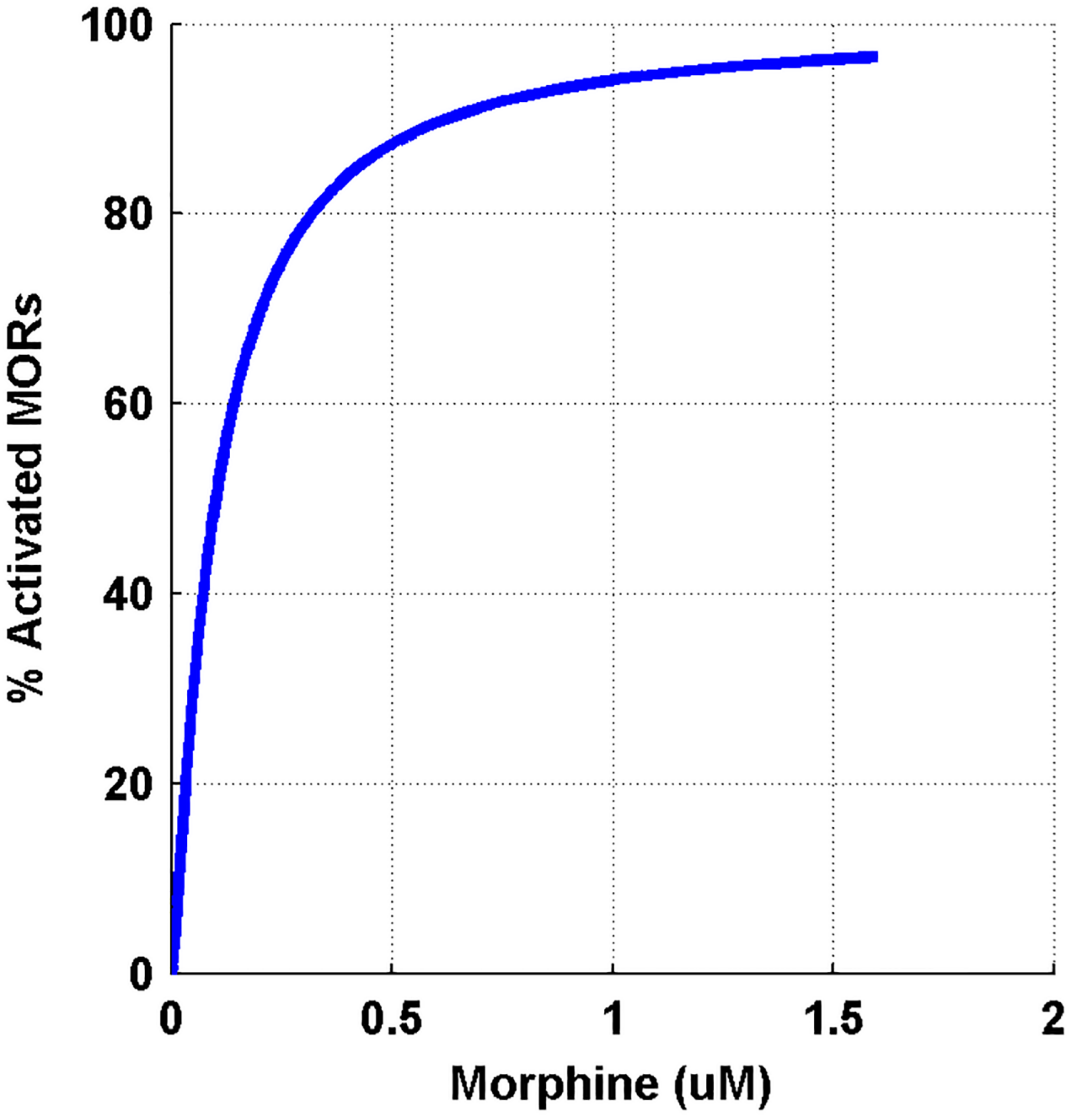
Shows the percentage of activated μORs versus different dose of morphine.

In the first part, μORs activation was simulated. By applying morphine, μORs of interneurons is activated. Elevation in the morphine dose activates the receptor more. Bourinet et al. showed that by activating μORs, calcium influx through N-type calcium channels attenuated [20]. Inhibition of calcium channels depends on the amount of μORs activation [21]. Less calcium entry into the interneuron leads to less inhibitory neurotransmitter release. Therefore, the Inhibitory Postsynaptic Currents (IPSCs) in the CA3 pyramidal neuron decreases. Capogna et al. denoted that inhibition of IPSC by morphine in CA3 pyramidal neuron depends on morphine dose. This dependency can be modeled in a hill function form [19]. Simulation result for activation of μORs with the variety of morphine concentration has been shown in Fig 3.

**Fig 4:**
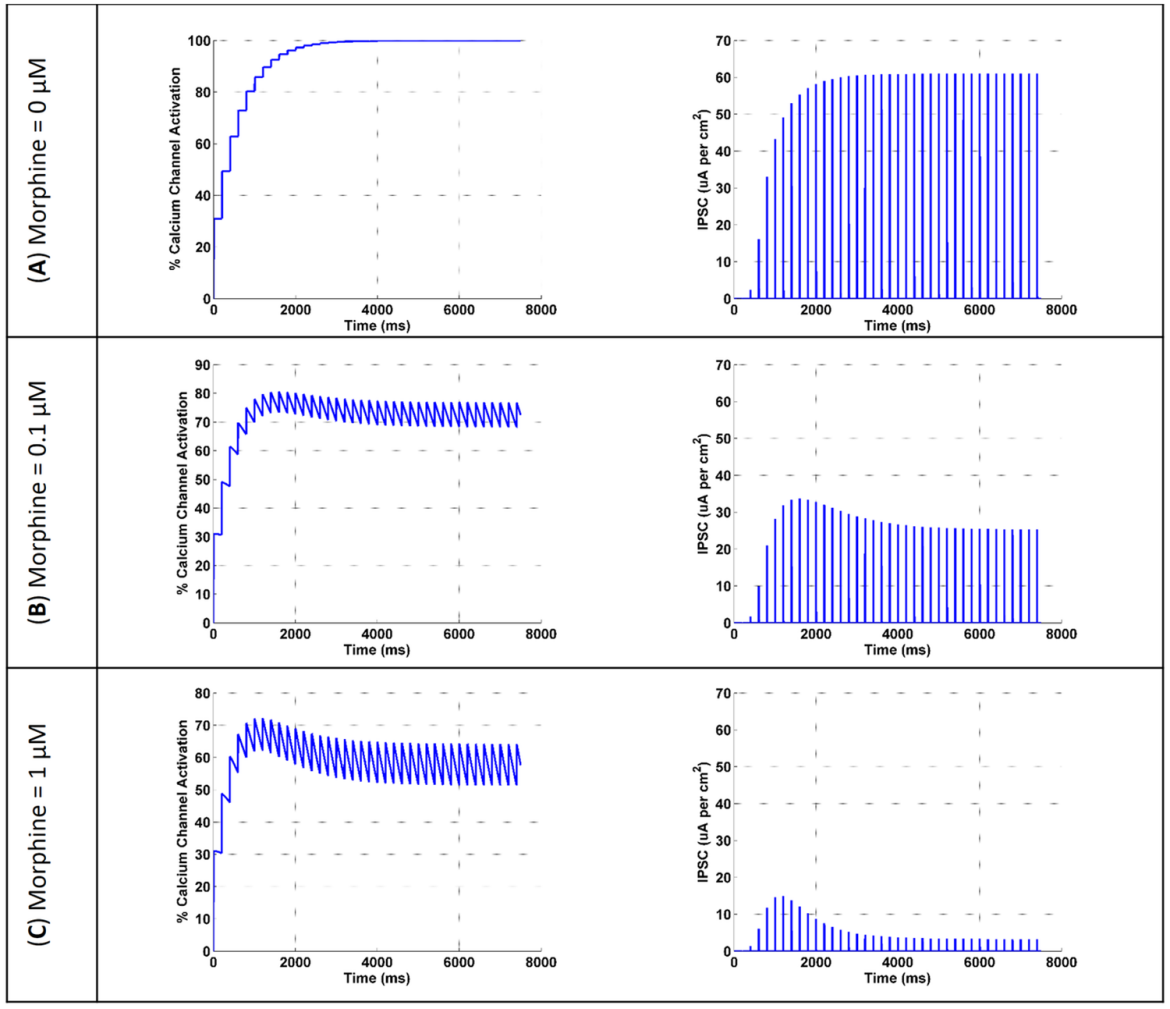
The activation of the interneuron calcium channel and the amplitude of the IPSC in the pyramidal neuron are shown for three different doses of morphine (μM). (A) morphine concentration considered to be 0, (B) shows the results for 0.1 μM concentration of morphine and (C) is the simulation results at 1 μM concentration of morphine.

μORs activation leads to inhibition of interneuron calcium channels activation. So, the amplitude of IPSC in pyramidal neuron attenuates. This process is shown in Fig 4 in response to different doses of morphine.

Fig 4A demonstrates that when there is not any morphine injection, all VGCCs are in an active state and the inhibitory current is at its maximum value. By applying 0.1 μM of morphine, VGCCs activation is attenuated to 75%, and IPSCs are decreased to 26 μA/cm^2 (Fig 4B). A further increase in the dose of morphine to 1 μM precedes to 59% activation of VGCCs and IPSCs are attenuated to approximately 8 μA/cm^2 (Fig 4C).

**Fig 5:**
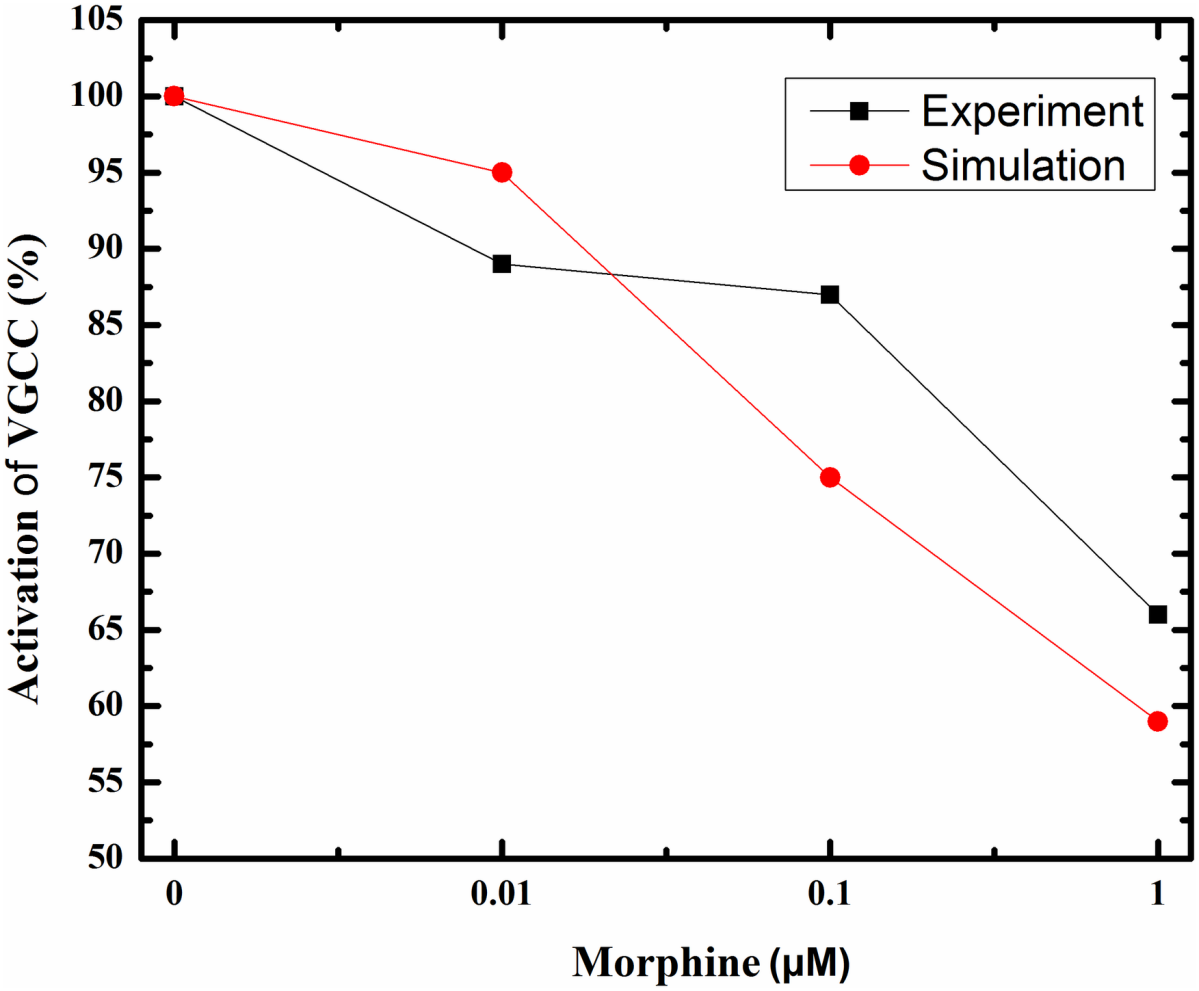
Activated VGCCs versus different doses of morphine (μM) in experimental conditions (red line) and simulation setup (black line) are described here.

**Fig 6:**
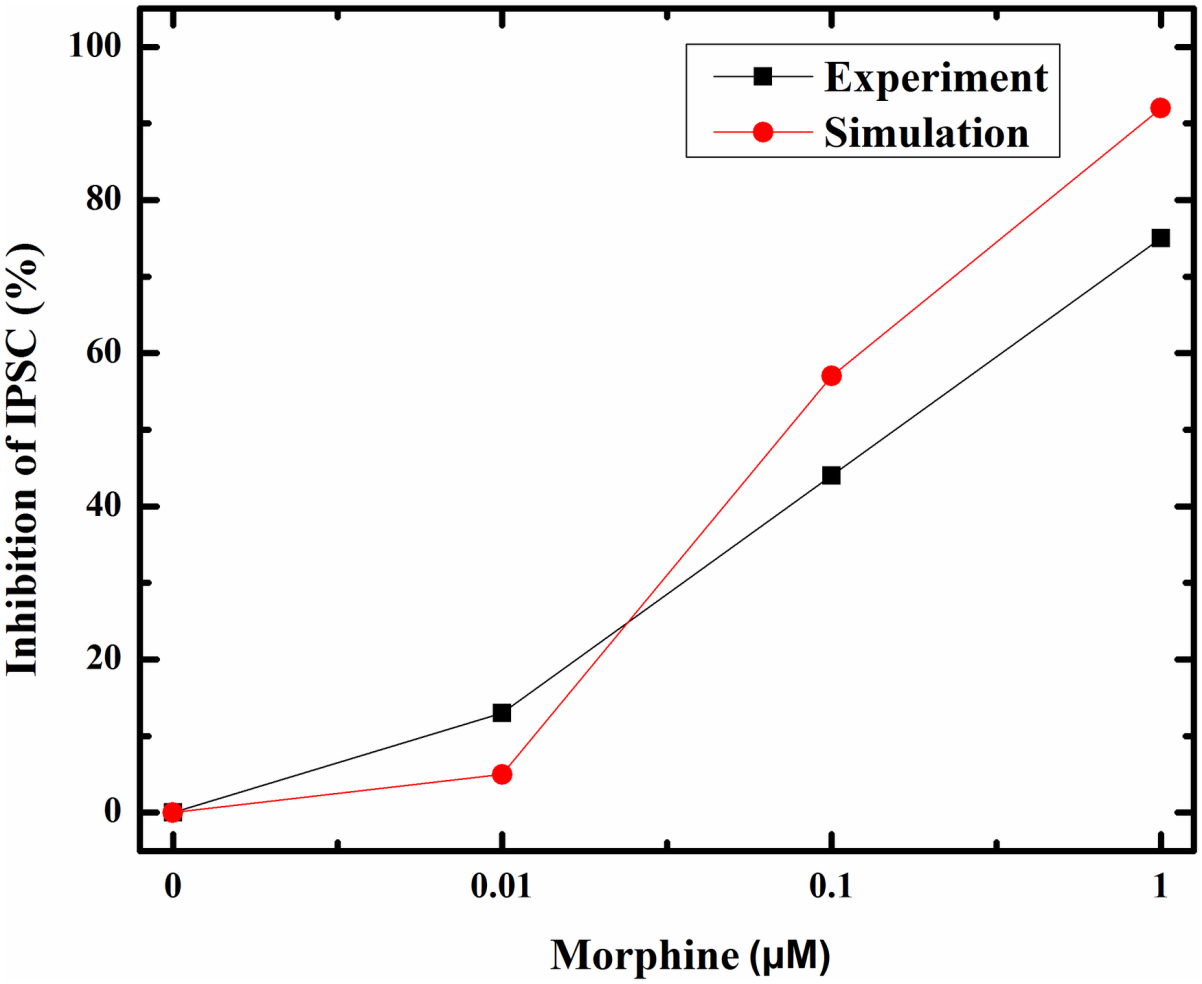
Inhibition of IPSC in response to different doses of morphine (μM) in experimental study (black line) and simulation condition (red line) are shown here.

Comparison of simulation results with the experimental findings for the activation of VGCCs and inhibition of the IPSC are denoted in Figs 5 and 6. During injection of 0.01 μM DAMGO (μOR agonist), calcium influx is decreased to 89% of its final value in the experiment [21]; simulation of the proposed model shows decrease in activated calcium channels to 95%. By increasing the dose of injected DAMGO to 0.1 μM, activated calcium channels attenuates to 87% in the experiment [21], and to 75% in the model. Further injection of DAMGO (1 μM) leads to more reduction in the calcium influx up to 66% in the experiment [21], and inhibition of activated channels decreases to 59% in the proposed model (Fig 5).

Inhibition of activated calcium channels in an inhibitory neuron can directly inhibit an inhibitory current on excitatory neurons based on Bertram’s assumption. In this part, inhibitory current on the CA3 pyramidal neuron in simulation compared with experimental data in Fig 6. Different doses of morphine decrease the inhibitory current in the pyramidal neuron by 75%, 44% and 13% for 1, 0.1 and 0.01 μM injection of morphine in the experimental condition, respectively [19]. Simulation results for the inhibitory current show decrease by 92%, 57% and 5% With 1, 0.1 and 0.01 μM of morphine, respectively. One can see a similar pattern in simulation results and experiments.

The model representing opioid receptor activation can relatively mimic the experimental results reported by Rhim et al. [21] and Capogna et al. [19].

### Normal condition

**Fig 7:**
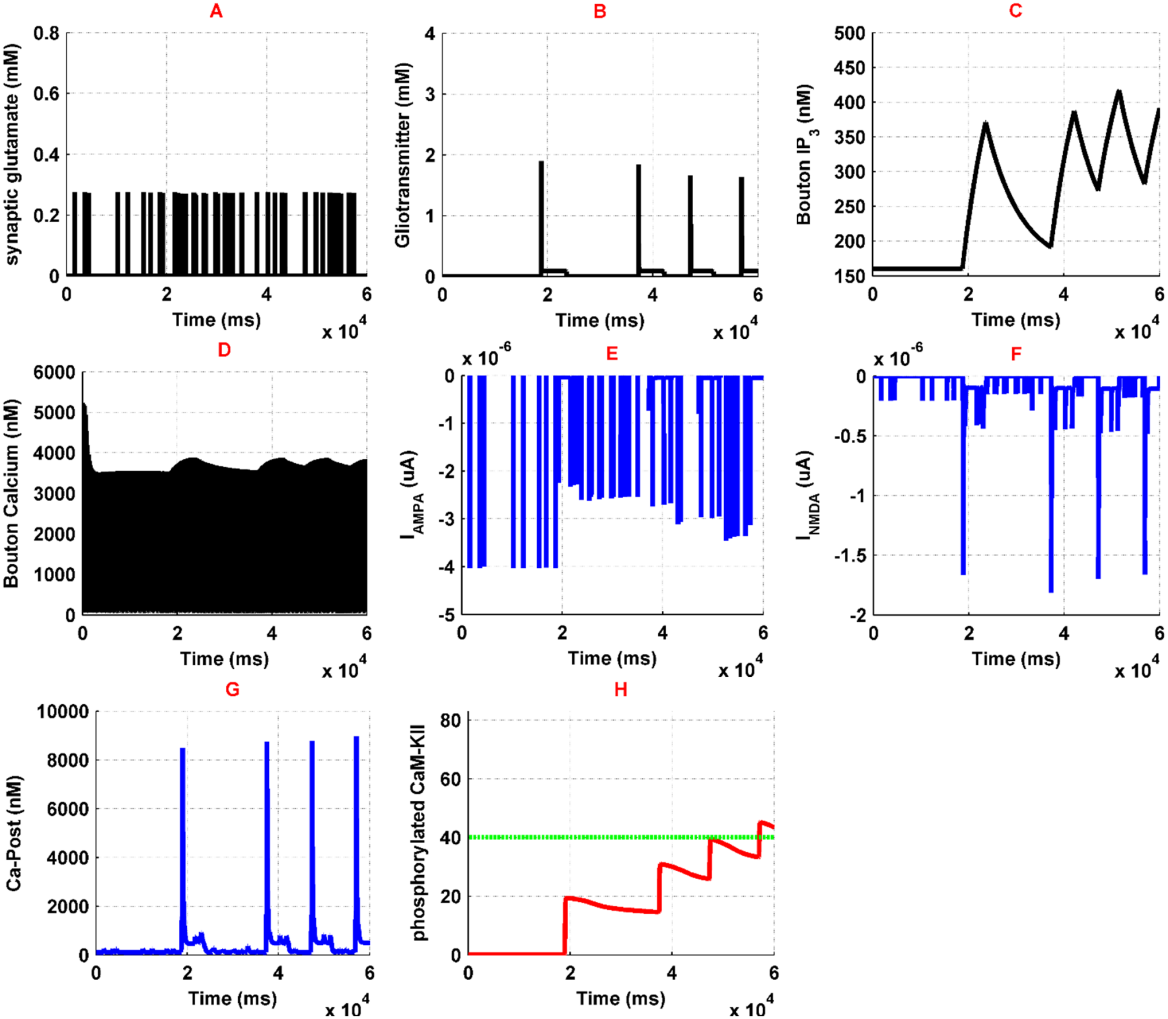
Simulation results for normal condition has been denoted here. Synaptic glutamate is shown in (A), gliotransmitter dynamics is denoted in (B), presynaptic IP3 variation and *Ca*^2+^ oscillations are shown in (C) and (D), respectively; AMPAR and NMDAR currents are denoted in (E) and (F), postsynaptic *Ca*^2+^ is shown in (G), phosphorylated CaMKII and NOs threshold are shown in (H) by red and green curves, respectively.

To compare opioid-induced LTP with the typical situation, first, we simulate the model for the normal state, where there is no contribution of opioid receptors activation in the simulations. Stimulation of CA3 pyramidal neuron and interneuron with a pulse train leads to the generation of APs. APs activate N-type VGCCs and the *Ca*^2+^ can enter into the neuron. Then, *Ca*^2+^ binds to the presynaptic *Ca*^2+^ sensors and triggers the glutamate release to the synaptic cleft (Fig 7A). In addition, the effect of Interneuron activation is an augmentation of *g*_*GABA*_ in the presynaptic pyramidal neuron which exerts a further inhibitory effect.

Synaptic glutamate can bind to astrocytic mGlurs and postsynaptic NMDA and AMPA receptors. By activation of astrocytic mGlurs, calcium concentration augments through IP3 production in astrocyte. Astrocytic *Ca*^2+^ oscillations lead to the gliotransmitter release, which affects both presynaptic and postsynaptic pyramidal neurons. Gliotransmitter release has been shown in Fig 7B. Gliotransmitter affects presynaptic mGlurs and leads to IP3 production which is shown in Fig 7C. Production of presynaptic IP3 leads to the enhancement of calcium concentration in the synaptic bouton (Fig 7D). Synaptic glutamate can bind to postsynaptic AMPARs and NMDARs. Activation of AMPARs (Fig 7E) depolarize postsynaptic membrane potential and convey to remove of NMDARs *Mg*^2+^ blocking, so NMDAR mediated current flows (Fig 7F). Therefore, *Ca*^2+^ influx to the postsynaptic neuron initiates. Postsynaptic *Ca*^2+^ oscillations are shown in Fig 7G. Enhancement of *Ca*^2+^ in the CA1 pyramidal neuron activates the CAMKII process which is able to augment AMPAR conductance and NO synthesis. In the Fig 7H, phosphorylated CaMKII is indicated by the red line and the threshold for the synthesis of NO with the green line. NO messenger can retrogradely diffuse from postsynaptic neuron to the presynaptic neuron and increases the sensitivity of *Ca*^2+^ sensors in the CA3 pyramidal neuron. Subsequently, there would be a further glutamate release in the synaptic cleft. This process acts as a positive feedback to inducing and maintaining the LTP in the synapse. Overall, this simulation shows a typical learning process in synaptic level.

### Pathological condition

**Fig 8:**
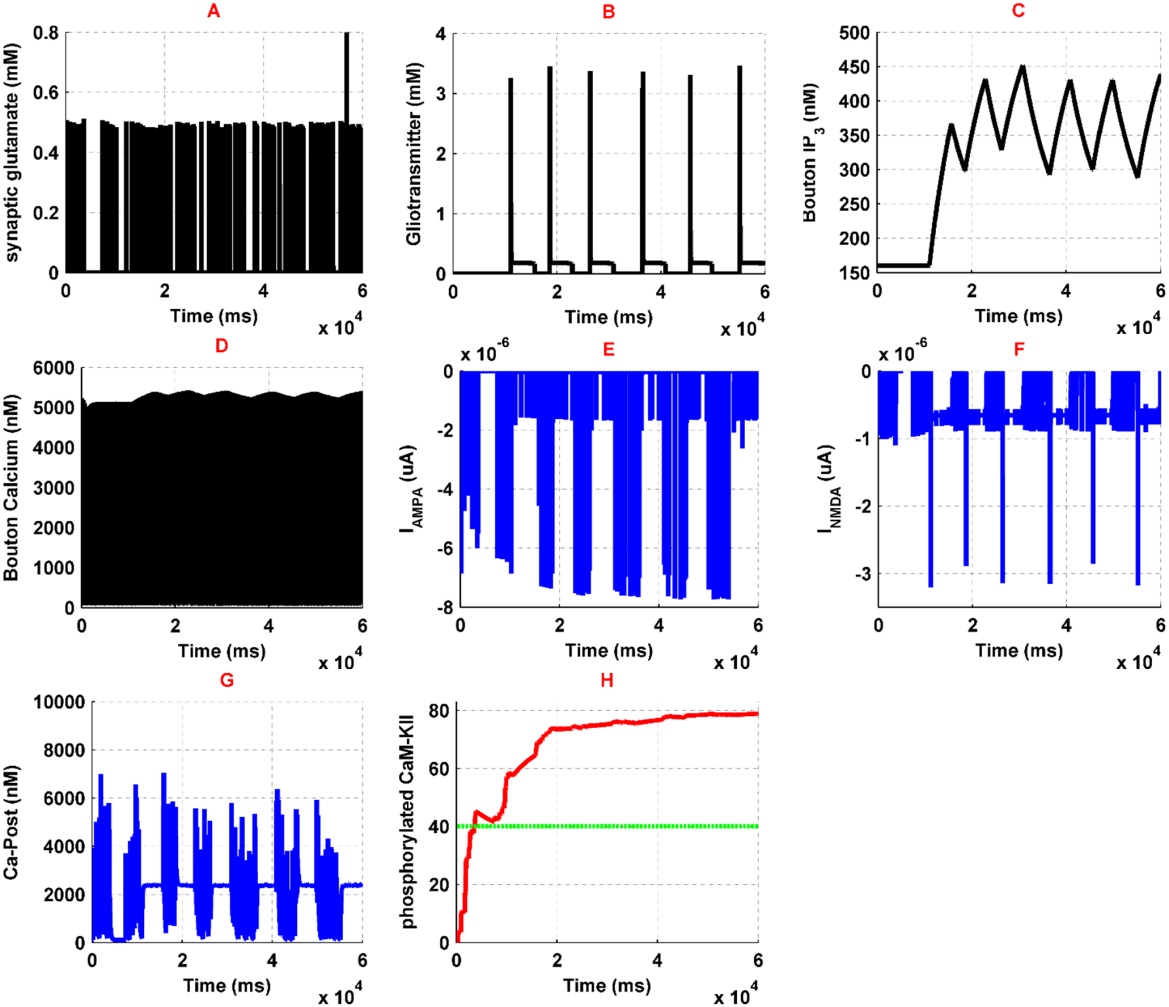
Results of the simulations in pathological conditions with an injection of 1 μM morphine have been denoted. Synaptic glutamate is shown in (A), gliotransmitter dynamics is denoted in (B), presynaptic IP3 variation and *Ca*^2+^ oscillations are shown in (C) and (D), respectively, postsynaptic *Ca*^2+^ is shown in (E), and finally, phosphorylated CaMKII and the threshold for NOs are shown in (F) with red and green lines, respectively.

Injection of 1 μM morphine activates presynaptic and postsynaptic μORs. Activation of presynaptic μORs inhibits the *Ca*^2+^ entrance into the interneuron by 40%, which is described in Fig 5. Therefore, IPSC of CA3 pyramidal neuron decreases to 8 μA/cm^2 due to fewer GABA releases by interneuron. Reduction of IPSC leads to more neuronal excitability. Consequently, synaptic glutamate increases and stimulates the astrocyte and postsynaptic neuron more than its normal condition. Synaptic glutamate has been shown in Fig 8A. As one can see, amplitude, frequency and the total amount of released glutamate by the presynaptic neuron is significantly increased. This increase is about six times larger than its normal condition and may lead to more excitation of both postsynaptic neuron and astrocyte. Furthermore, gliotransmitter release by astrocyte is enhanced by 250% of its normal state which is described in Fig 8B. Gliotransmitter increase is due to further activation of astrocytic mGlurs and enhancing calcium oscillations of the astrocyte. Binding gliotransmitter to presynaptic mGlurs acts as a positive feedback and results in IP3 production and increase of presynaptic calcium, which are denoted in Fig 8C and 8D, respectively. As it has been shown in the Fig 8C, IP3 production is enhanced in the presynaptic neuron, which can suggest the increase of calcium in synaptic bouton. Furthermore, since glutamate reuptake is attenuated in the presence of opioids, we modeled it by decreasing the reuptake ability of glutamate by astrocyte down to 50% of a typical situation based on experimental observations.

On the other hand, the presence of opioids affects NMDAR activation by inhibiting *Mg*^2+^ blocking and by increasing conductance of NMDA receptors. Due to experimental research, *Mg*^2+^ blocking of NMDARs modified in which δ (the relative electrical distance of the binding site of *Mg*^2+^ from the outside of membrane) increased by 11% and *K*_0_ (dissociation constant at 0 mv) augmented by 4.8 fold of its normal value. Besides that, morphine can increase NMDARs voltage independent conductance by 15% averagely, by Protein Kinase C (PKC) mechanisms, which has been applied in the model.

Generally, in the pathological conditions, postsynaptic calcium’s entry to the neuron has been denoted in Fig 8G, which shows a significant increase in comparison to the normal state. This amplification of calcium oscillations is due to the enhancement of NMDARs activation (Fig 8F). NMDAR related current increases because it receives more glutamate from the presynaptic neuron and more gliotransmitter from astrocyte. Besides that, activation of the μORs enhance conductance of the NMDARs through the previously mentioned mechanisms. Calcium entry to the postsynaptic neuron causes phosphorylation of CaMKII which is shown in Fig 8H. Phosphorylation of CaMKII is followed by NO synthesis. Synthesized NO retrogrades to the presynaptic neuron and enhances the binding ability of glutamate vesicles to calcium ions. Besides that, enhancing calcium entry to the postsynaptic neuron also leads to an increase in AMPAR conductance by approximately 30% in pathological condition. Generally, with the assumption that enhancement of the CaMKII phosphorylation, AMPARs conductivity, glutamate, and gliotransmitter are the main traces of the induction of LTP in the synapse, morphine injections appear to lead to pathological memory.

**Fig 9:**
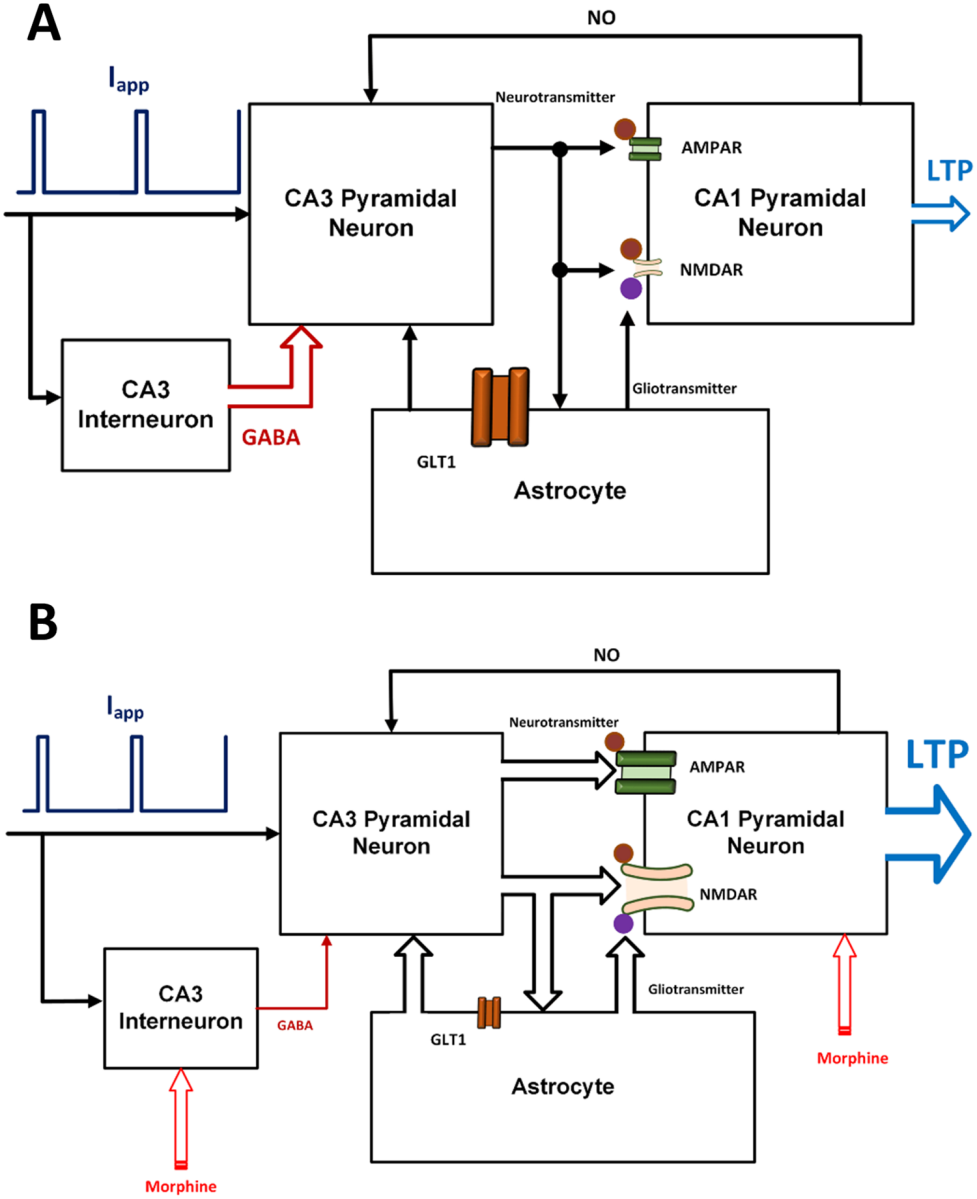
A comparison between the normal (A) and the pathological (B) conditions is shown.

It has been shown that opioid presence leads to LTP production. Fig 9 compares normal and pathological conditions in two block diagrams. Under normal conditions, the interneuron regulates the activity of the presynaptic pyramidal neuron, and inhibitory current is significant. Also, astrocytic transporters work normally (Fig 9A). In the pathological conditions, the inhibitory current decreases and the amount of neurotransmitters and gliotransmitters increases. Also, by applying morphine, the sensitivity of the postsynaptic NMDARs to the transmitters enhances. Therefore, more calcium enters into the neuron. The increase in calcium conveys to CaMKII phosphorylation and thus NO is synthesized. Also, AMPAR’s conductance increases due to CaMKII phosphorylation process (Fig 9B).

### Comparing the effect of different factors on pathological LTP induction

**Fig 10:**
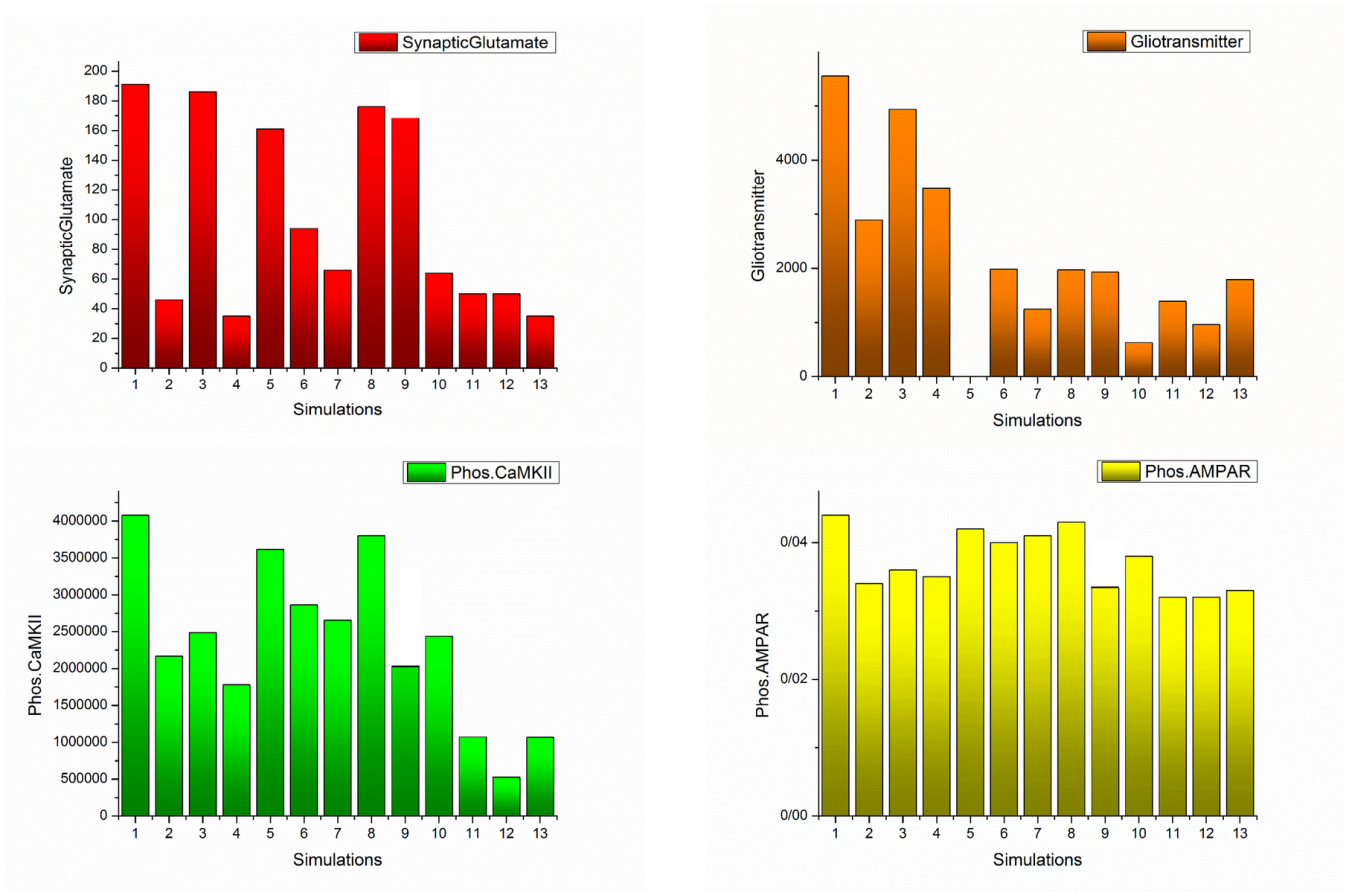
Box and whisker plot for different simulations have been described here. Redline shows the median of the signals, ‘+’ sign represents the mean value of each signal, blue line demonstrates that 25%-75% of data are included in this range, and finally black line shows the range that 9%-91% of the dada are included. Panel A shows the synaptic glutamate, Panel B denotes the gliotransmitter, Panel C describes the phosphorylated CaMKII, and Panel D represents the Phosphorylated AMPARs. In the first simulation, all of the synaptic components are affected by morphine and the simulation of 13^th^ indicates normal mode. In the second simulation, the effect of morphine on presynaptic neurons has been neglected. In the third simulation, postsynaptic μORs are assumed to be inactive. In the fourth simulation, morphine only affects astrocyte. In the fifth simulation, the release of the gliotransmitter is considered zero. In the sixth simulation, it is assumed that despite the effect of morphine on all the components of the synapse, astrocytic GLTs act normal. In the seventh simulation, under conditions where morphine affects all of the synaptic components, the activity of astrocytic transporters is stimulated to be 50% greater than normal. In the eighth simulation, the activity of astrocytic mGlurs is considered zero. The ninth simulation shows that astrocytic mGlurs and postsynaptic μORs are not active. In the tenth simulation, astrocytic transporters are stimulated 50% more than normal, and astrocytic mGlurs have been deactivated. In the eleventh simulation, by stimulating astrocytic transporters, NMDA receptors have a normal synaptic effect. In the 12^th^ simulation, astrocytic mGlurs have been deactivated, astrocytic transporters are stimulated, and NMDARs act as normal.

In the previous simulations, it has been demonstrated that morphine injection conveys to pathological LTP induction. In this section, the effect of the different factors on the induction of pathological LTP is considered and significant parameters are specified. Essential elements can be seen as drug targets to prevent the formation of pathological memory in the use of opioids. The presence of 1 μM morphine in synapse can enhance CaMKII phosphorylation process, synaptic glutamate, gliotransmitter release, and AMPAR conductance. These variables are considered as *monitoring factors* for morphine effects on the synapse. Monitoring elements can be seen as a trace of LTP induction. Fig 10 shows the effects of different parameters on monitoring factors through the simulations shown in the next section.

#### Simulation 1

In the presence of 1μM morphine, monitoring factors are shown in Fig 10. In Simulation 1 morphine affects presynaptic, postsynaptic and astrocyte. Therefore, presynaptic and postsynaptic μORs are in the active state, and astrocytic glutamate transporters coefficient is half of the normal conditions, which indicates the effect of morphine on astrocyte.

Comparing these results with normal conditions (Simulation 13) infers that the average amount of synaptic glutamate is five times higher than normal. The average amount of gliotransmitter is 4.5 times the normal state. The mean amount of phosphorylated CaMKII and AMPARs are 4 and 1.3 times higher than the normal level. Overall, in the presence of morphine, all of the monitoring factors are high, which can be indicative of LTP induction.

#### Simulation 2

In this simulation, we want to show how the disinhibitory effect of morphine affects monitoring factors. Here, we disable the presynaptic opioid receptors and morphine only affects the postsynaptic μORs and astrocytic GLT1s.

Simulation results denote that despite a significant reduction in synaptic glutamate, other monitoring factors are still high and pathological LTP is still present. This means that, despite the importance of disinhibitory mechanism in inducing LTP, preventing it cannot block pathological memory.

#### Simulation 3

In this simulation, the postsynaptic μORs activity is inhibited. however, presynaptic neurons and astrocyte are under the influence of morphine. The results indicate that, despite the attenuation of CaMKII phosphorylation and the reduction of AMPAR conductance, the amount of glutamate and gliotransmitter are still highand the probability of formation of pathologic memory is high.

#### Simulation 4

In this section, morphine is thought to only affect astrocyte. Therefore, presynaptic and postsynaptic μORs are inhibited. In this case, the synaptic glutamate is approaching a level that is normal. In addition, phosphorylation of CaMKII and AMPARs has been significantly reduced. But it seems that due to the significant release of glutamate in the synapse. LTP occurs. This simulation describes that astrocyte plays a fundamental role in the induction of pathological LTP, so it is necessary to carefully examine its role in the following sections.

#### Simulation 5

In this simulation, it has been assumed that morphine affects all the components of the synapse, but astrocyte cannot release the gliotransmitter. Here astrocyte does not have any effect on presynaptic and postsynaptic neurons. The results suggest that inhibiting gliotransmitter release cannot disrupt the pathological memory formation.

#### Simulation 6

In this simulation, the role of astrocytic transporters in pathological memory induction has been investigated. Therefore, under the conditions of the first simulation, it is assumed that astrocyte transports are active as normal and their activity is not affected by the presence of morphine. The results show that normal activity of transporters cannot prevent the formation of pathologic memory.

#### Simulation 7

In this section, when morphine affects all the components of the synapse, the activity of astrocytic transporters enhanced by 50% more than their standard value. In this case, glutamate and gliotransmitter values have gone to normal, and CaMKII phosphorylation has been significantly reduced. This simulation shows that up-regulation of astrocytic transporters can be used as an agent to prevent the pathological LTP formation.

#### Simulation 8

In this simulation, the effect of astrocytic mGlur has been investigated. To this purpose, astrocytic mGlurs are inhibited in a situation where morphine affects all of the synaptic components. It can be seen that in this case, with the exception of the gliotransmitter which is in the normal range, the rest of the monitoring factors are high.

#### Simulation 9

In this simulation, postsynaptic opioid receptors and astrocytic mGlur are supposed to be inhibited. The results state that it is still possible to develop pathological memory.

#### Simulation 10

In this simulation, astrocytic mGlurs is inhibited and its transporters are stimulated 50% more than normal. Opioid receptors for presynaptic and postsynaptic neurons are still active. Results indicate that in spite of a significant drop in glutamate and gliotransmitter, the rest of the monitoring factors were not significantly reduced.

#### Simulation 11

In this simulation, postsynaptic opioid receptors have been disabled and astrocytic GLTs are stimulated 50% more than normal. The results show that in the presence of morphine, all of the monitoring factors are in normal range.

#### Simulation 12

In this simulation, postsynaptic opioid receptors and astrocytic mGlurs are supposed to be inhibited and astrocytic transporters are stimulated 50% higher than normal. In this case, monitoring factors are found to be close to the normal value, and even take less than normal values.

#### Simulation 13

This simulates the normal state in which it is assumed that morphine is not present in the synapse.

## Discussion

This paper presented a computational model in CA3-CA1 region of the hippocampus at the synaptic level. Here we extended and improved previously introduced models by different researchers [22, 23, 34, 41, 42, 45, 46, 48–53] to represent opioid-induced synaptic plasticity in the hippocampus. To best of our knowledge, this is the first model that has the ability to be used for computational analysis of opioid-induced synaptic plasticity. Using computational models allows researchers to analyze the effect of various parameters simultaneously without concerning about the interfering effects of different medical agents in experiments.

**Fig 11:**
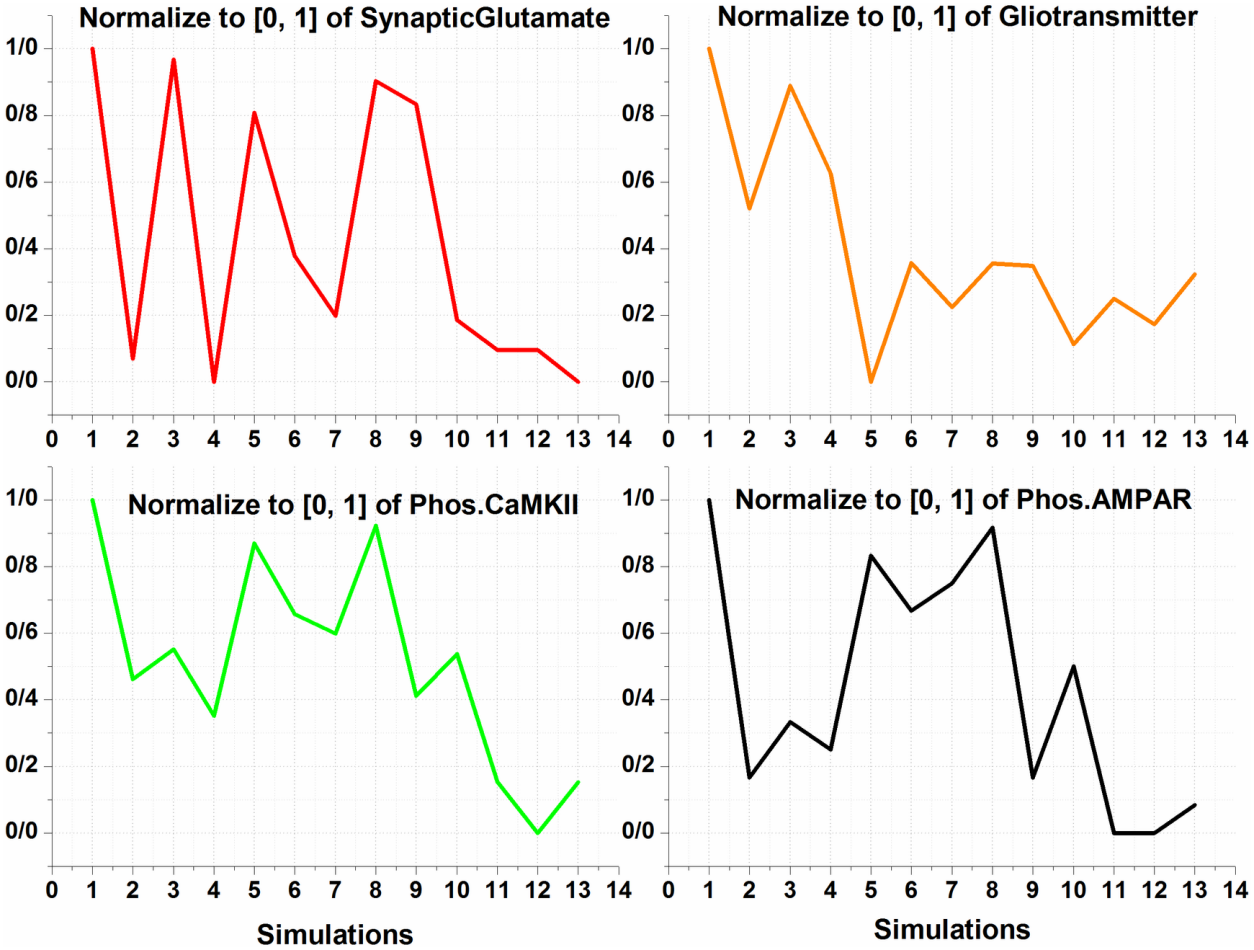
Normalized graph of monitoring factors for different simulations has been described here.

Our model adequately shows that opioids presence can induce LTP in affected synapses. Here, it is assumed that amounts of synaptic glutamate, gliotransmitter, phosphorylated CaMKII and AMPARs are footprints of LTP induction in the synapse. In fact, these factors are considered to be parameters which can lead to LTP induction, and we called them as monitoring factors. To discuss the results of the simulations, the normalized values for monitoring factors are depicted in Fig 11.

As previously stated, the first simulation shows that morphine affects all the components of the synapse and the thirteenth simulation indicates the normal mode. It has been shown in experiments that the presence of morphine increases the density of AMPARs, synaptic glutamate and calcium signaling in affected neurons [9, 54]. These changes, which describe the main features of pathological LTP and memory formation, are seen in the results of the first simulation.

In the second simulation, presynaptic opioid receptors are inhibited. As a result, release of glutamate is normal, but the traces of pathological LTP is still existed. The results of this simulation indicate that increasing the activity of postsynaptic NMDARs and the effect of morphine on astrocyte are factors that contribute to the formation of pathologic memory.

In the 3^rd^ simulation, postsynaptic μORs have been inhibited, indicating that postsynaptic NMDARs exhibit normal activity. In this case, the monitoring factors have significant amounts. It infers that injection of NMDAR antagonist or inhibition of postsynaptic opioid receptors is effective in preventing the induction of pathologic LTP, but it is not enough.

In fact, experimental results have also shown that NMDARs play an essential role in inducing opioid-related neural plasticity [38, 55–57]. Furthermore, it has been shown that NMDAR antagonist injections are effective in relapse weakening; however, it cannot completely eliminate drug-seeking behavior [58, 59]. Based on the results we have obtained in the simulations, it is true that NMDARs are the main actors in the formation of pathological memories, but the role of other parameters should not be ignored.

In the fourth simulation, only astrocyte is affected by morphine, and presynaptic and postsynaptic opioid receptors are inhibited. In this case, glutamate is found to be in normal range, but the amounts of CaMKII and AMPAR phosphorylation are still significant. As a result, it can be concluded that, regardless of the role of astrocyte in synapse, the pathologic LTP cannot be controlled. Some researchers believe that astrocyte dysfunction can lead to addiction [28, 30]. Thus, it can be concluded that without the manipulation of astrocyte function, we may not be able to inhibit the pathological LTP induction.

In addition, in the 5^th^ and 6^th^ simulations, it was observed that preventing the release of gliotransmitter and having normal GLTs cannot influence the formation of pathological LTP.

In the 7^th^ simulation, astrocytic transporters have been stimulated by 50% more than normal. It can be seen that despite the attenuation of synaptic glutamate and gliotransmitter, other monitoring factors are still high. So, stimulating astrocytic GLTs can be effective, but is not sufficient for preventing pathological LTP. Now the important question is: is it possible to up-regulate astrocytic glutamate transporters empirically? The answer is yes. It has recently been shown that up-regulation of astrocytic transporters is possible and can even undermine the induction of LTP in the synapse [60].

In the 8^th^ simulation, astrocytic mGlurs are inhibited. This mode describes that the agonist-dependent IP3 production is inhabited in astrocyte. According to the results obtained, this mode alone cannot be used as an effective parameter.

In the 9^th^ simulation, astrocytic mGlurs and postsynaptic μORs are simultaneously inhibited. In this case, synaptic glutamate is high, phosphorylated CaMKII is almost halved, and the amount of phosphorylation of AMPARs and released gliotransmitter are in normal range. It can be noticed that blocking the agonist-dependent IP3 production and the use of NMDAR antagonist can undermine the pathological LTP. In this case, the only remaining problem is to reduce the amount of synaptic glutamate that can also regulate CaMKII phosphorylation.

In the 10^th^ simulation, activity of astrocytic mGlurs is inhibited, and GLTs are stimulated to be 50% more than normal level. In this case, glutamate and gliotransmitter are in the normal range, and the other monitoring factors are almost halved. Therefore, it can be concluded that manipulation in the activity of astrocyte can be effective in preventing the formation of pathological LTP.

The 11^th^ simulation describes the case in which astrocyte transporters are stimulated, and postsynaptic μORs are inhibited. In this case, all monitoring factors fall into the normal range. It means that stimulating GLTs and injection of NMDAR antagonists can stop the formation of pathological LTP.

In the 12^th^ simulation, in addition to inhibiting the activity of postsynaptic opioid receptors and stimulating GLTs, we also disable astrocytic mGlurs. In this case, monitoring factors are alose to normal and even some of them have values that are less than normal. It can be concluded that in this case the possibility of the formation of pathological memory is completely eliminated.

Overall, by summerizing all the simulations, it can be suggested that an optimal method for coping with the formation of pathological memory can be the stimulation of astrocyte GLTs and the injection of NMDAR antagonists.

In this paper, some assumptions are made to simplify the modeling process. Dynamics has not been considered for the release of inhibitor neurotransmitter from the interneuron to the pyramidal neuron in the presynaptic layer. Such an approach has been implemented in some computational models. It is also assumed that interneuron and astrocyte do not interact. Investigation of this interaction and its role in the induction of the synaptic plasticity can be considered in the future works. Also, in this paper, as with some computational models, the diffusion process for glutamate and gliotransmitter is not considered. It seems that focusing on this process in models that deal with a large number of neurons (network level) can be effective, but in our model, it will not have much impact on the results. On the other hand, we assume that some factors are footprints of LTP induction in the synapse, such as the concentration of glutamate and gliotransmitter, and phosphorylation of CaMKII and AMPARs. Considering the effect of LTD in synapse can extend the model. This goal may be accomplished through the use of the STDP process as a learning rule in synapse.

## Conclusion

Opioids such as morphine can lead to the induction of LTP at the synaptic level, which may result in an abnormal memory formation process leading to addiction. This pathological memory can be seen as the key trigger for relapse in addicted patients. Suppression of LTP induced by opioids may lead to diminishing relapse in withdrawal period in addicted subjects. Since changing different parameters and evaluating various types of effects in memory formation process at the synaptic level can be difficult and even practically impossible in experimental conditions, a detailed mathematical model can help us to overcome empirical obstacles and difficulties to some extent. This paper, presented a biophysically suggested and supported computational model for opioid-induced memory formation process. The proposed model is at the synaptic level and the role of astrocyte is considered as the third component of synapse. The proposed model is capable of describing the pathologic LTP induction process and can be helpful in addiction-related research. Here, we found that astrocytic transporters play a fundamental role in the induction of pathological drug-related memories. Simulation results show that up-regulating astrocytic transporters, omitting astrocytic mGlurs and applying NMDARs antagonist can completely prevent pathological LTP. We also suggest that the up-regulation of GLTs and injection of NMDAR antagonist can be used as an optimal way to prevent the occurrence of pathological LTP. Overall, we suggest that astrocyte has a prominent role in addiction-related memories and its function must be considered precisely for preventing abnormal memories. This approach may help researchers to inhibit relapse in the future.

## Appendix

### Description of functions

#### Presynaptic neurons

Presynaptic APs is governed by H-H type formulation:

$$\frac{dV_{pre}}{dt}=f_{Pyr}(I_{app},{\text{I}}_{Na},{\text{I}}_{K},{\text{I}}_{L},{\text{I}}_{GABA_{A}})=I_{app}-36n^{4}(V_{pre}+82)-120m^{3}h(V_{pre}-45)-0.3(V_{pre}+59.4)-I_{GABA}$$

$$\frac{dV_{Int}}{dt}=f_{Int}(I_{app},I_{Na},I_{K},{\text{I}}_{L})=I_{app}-36n^{4}(V_{Int}+82)-120m^{3}h(V_{Int}-45)-0.3(V_{Int}+59.4)$$

$$\frac{dx}{dt}=\alpha_{x}(1-x)-\beta_{x}x,\alpha_{n}=\frac{0.01(-{\text{V}}_{pre}-60)}{\text{exp}(\frac{-{\text{V}}_{pre}-60}{10})-1},\alpha_{m}=\frac{0.1(-{\text{V}}_{pre}-45)}{\text{exp}(\frac{-{\text{V}}_{pre}-45}{10})-1},\alpha_{h}=0.07\text{exp}(\frac{-{\text{V}}_{pre}-70}{20}),\beta_{n}=0.125\text{exp}(\frac{-{\text{V}}_{pre}-70}{80}),\beta_{m}=4\text{exp}(\frac{-{\text{V}}_{pre}-70}{18}),\beta_{h}=\frac{1}{\text{exp}(\frac{-{\text{V}}_{pre}-40}{10})+1}$$ (A-1)

Here *V*_*pre*_ is presynaptic membrane potential for the pyramidal neuron, *V*_*Int*_ is the potential of presynaptic interneuron, *I*_*app*_ is the externally applied current, g_*K*_, g_*Na*_ and g_*L*_ are potassium, sodium and leak conductance respectively, *V*_*N*_, *V*_*K*_, and *V*_*L*_ are sodium, potassium and leak reversal potentials respectively. Third equation denotes gating variables for ionic currents, x can be substituted by m, n, h for gating variable of *Na*^+^ activation, *K*^+^ activation, and *Na*^+^ inactivation respectively.

Calcium oscillations can be represented by [22]:

$$\frac{dc_{Fast}}{dt}=f_{Fast}({\text{I}}_{Ca},{\text{I}}_{PMCa},{\text{I}}_{PMleak},b_{n})=-\frac{I_{Ca}A_{btn}}{z_{Ca}FV_{btn}}+J_{PMleak}-\frac{I_{PMCa}A_{btn}}{z_{Ca}FV_{btn}}$$ (A-2)

Where calcium current through N-type channels represents by *I*_*Ca*_, *A*_*btn*_ is neuron bouton’s surface area, *z*_*Ca*_ is the calcium ion valence, *V*_*btn*_ is the neuron bouton’s volume, *F* is faraday’s constant. *J*_*PMleak*_ is the leak current from extracellular space into Bouton, *I*_*PMCa*_ denotes calcium current due to ATPase pump which pumps extra calcium out of the cell and *I*_*Ca*_ is the calcium current due to N-type VGCCs and is governed by:

$$J_{PMleak}=\nu_{leak}(c_{ext}-c_{i}),I_{PMCa}=\nu_{PMCa}\frac{c_{i}^{2}}{c_{i}^{2}+K_{PMCa}^{2}},I_{Ca}=\rho_{Ca}\;m_{Ca}^{2}\;g_{Ca}(V_{pre}(t)-V_{Ca})$$ (A-3)

Here, *ν*_*leak*_, *c*_*ext*_ and *c*_*i*_ are the maximum leak of calcium through the membrane, extracellular calcium concentration, and intracellular calcium concentration respectively. *ν*_*PMCa*_ is maximum PMCa current and *K*_*PMCa*_ is the calcium concentration at which *ν*_*PMCa*_ is halved. Here, *ρ*_*Ca*_ denotes N-type calcium channel density on the membrane of neuron Bouton, *g*_*Ca*_ is the single channel’s conductance. In the above equation assumed that N-type channel has two gates. *m*_*Ca*_ is the opening probability of a single gate and can be shown by:

$$\frac{dm_{Ca}}{dt}=\frac{m_{Ca}^{\infty }-m_{Ca}}{\tau_{m_{Ca}}},m_{Ca}^{\infty }=\frac{1}{1+\text{exp}((V_{m_{Ca}}-V_{m})/k_{m_{Ca}})},V_{Ca}=\frac{RT}{z_{Ca}F}\text{ln}(\frac{c_{ext}}{c_{i}^{rest}})$$ (A-4)

Moreover, $\tau_{m_{Ca}}$ is the time constant where *m*_*Ca*_ approaches to $m_{Ca}^{\infty }$ which is the Boltzmann function with $V_{m_{Ca}}$ and $k_{m_{Ca}}$ as the half-activation voltage of N-type calcium channel and the slope factor of N-type channel activation respectively. *V*_*Ca*_ shows reversal potential of *Ca*^2+^ the ion. Here, *R* is the real gas constant, *T* denotes absolute temperature, extracellular calcium concentration is shown by *c*_*ext*_ and intracellular calcium concentration at rest is denoted by $c_{i}^{rest}$. Parameters related to presynaptic calcium dynamics can be found in Tewari and Majumdar’s paper [22].

Modeling slow calcium oscillations achieved by using Tewari and Majumdar’s modified Li-Rinzel model which can describe by [22]:

$$\frac{dc_{Slow}}{dt}=f_{Slow}({\text{J}}_{chan},{\text{J}}_{ERpump},{\text{J}}_{ERleak})=-J_{chan}-J_{ERpump}-J_{ERleak}$$ (A-5)

Here *J*_*chan*_ denotes calcium influx from ER into cytosol through the IP3 receptor, *J*_*ERpump*_ is the pumped calcium from intracellular space to ER and *J*_*ERleak*_ indicates leaked calcium ions from ER into intracellular space. Equations that describe these three types of *Ca*^2+^ flux are:

$$J_{chan}=c_{1}\nu_{1}m_{\infty }^{3}n_{\infty }^{3}q^{3}(c_{i}-c_{ER}),J_{ERpump}=\frac{v_{3}c_{i}^{2}}{k_{3}^{2}+c_{i}^{2}},J_{ERleak}=c_{1}\nu_{2}(c_{i}-c_{ER})$$

$$\frac{dc_{ER}}{dt}=-\frac{1}{c_{1}}\frac{dc_{slow}}{dt},\frac{dp}{dt}=\nu_{g}\frac{g_{a}^{0.7}}{k_{g}^{0.7}+g_{a}^{0.7}}-\tau_{p}(p-p_{0}),\frac{dq}{dt}=\alpha_{q}(1-q)-\beta_{q}q$$ (A-6)

Where *c*_*ER*_ is the *Ca*^2+^ concentration in ER, *c*_1_ is the ratio of ER volume to bouton volume, *ν*_1_ denotes maximal flux rate of *IP*_3_ receptors, *ν*_2_ shows maximal leak of *Ca*^2+^ from ER to intracellular space, *ν*_3_ is the maximal SERCA (Sarco Endoplasmic Reticulum ATPase) pump rate, intracellular *IP*_3_ concentration is demonstrated by *p* finally *g*_*a*_ and *q* are Extrasynaptic glutamate concentration and fraction of activated *IP*_3_ receptors respectively. Also, *m*_∞_, *n*_∞_, *α*_*q*_ and *β*_*q*_ can be denoted by:

$$m_{\infty }=\frac{p}{p+d_{1}},n_{\infty }=\frac{c_{i}}{c_{i}+d_{5}},\alpha_{q}=a_{2}d_{2}\frac{p+d_{1}}{p+d_{3}},\beta_{q}=a_{2}c_{i}$$ (A-7)

Here, *d*_1_ and *d*_3_ are the *IP*_3_ dissociation constant, *d*_2_ and *d*_5_ are the inhibitory *Ca*^2+^ dissociation and activation *Ca*^2+^ dissociation constants respectively. *a*_2_ is the inhibitory *Ca*^2+^ binding constant. Further information about the equations can be found in Tewari and Majumdar’s paper [22].

#### Astrocyte *Ca*^2+^ dynamics

Equations which describe *Ca*^2+^ oscillations in astrocyte are [46]:

$$\frac{dc_{Astro}}{dt}=f_{Astro}(J_{chan,a},J_{pump,a},J_{leak,a})=-J_{chan,a}-J_{pump,a}-J_{leak,a}$$ (A-8)

$$J_{chan,a}=c_{1,a}r_{c_{a}}m_{\infty }^{3}n_{\infty }^{3}h_{\infty }^{3}(c_{Astro}-c_{ER,a}),J_{pump,a}=v_{ER}\frac{c_{Astro}{}^{2}}{c_{Astro}{}^{2}+K_{ER}^{2}},J_{leak,a}=c_{1,a}r_{L}(c_{Astro}-c_{ER,a})$$

Where *c*_*Astro*_ denotes *Ca*^2+^ concentration in intracellular space of astrocyte, *J*_*chan*,*a*_ is *Ca*^2+^ flux from ER to the intracellular space due to *IP*_3_ binding to ER’s*IP*_3_*R*, *J*_*pump*,*a*_ is the *Ca*^2+^ removing from intracellular space by SERCA pump, *J*_*leak*,*a*_ is the *Ca*^2+^ leak from ER to intracellular space, *c*_1,*a*_ is the ratio of ER volume to cytosol volume,$r_{c_{a}}$ is the maximum rate of *Ca*^2+^ flux by *IP*_3_*R*, $m_{\infty }^{3}n_{\infty }^{3}h_{\infty }^{3}$ is the opening probability of *IP*_3_*R* vesicles, *c*_*ER*,*a*_ shows *Ca*^2+^ concentration in ER, *ν*_*ER*_ is the highest rate of *Ca*^2+^ uptake by ER, *K*_*ER*_ denotes intracellular calcium affinity of SERCA pump and *r*_*L*_ shows maximum rate of *Ca*^2+^ leak from ER. Equations of *IP*_3_ dynamics in astrocyte can be written as follows:

$$\begin{array}{l} \frac{dp_{a}}{dt}=v_{\beta }Hill\left(g^{0.7},K_{R}\left(1+\frac{K_{p}}{K_{R}}Hill(C,K_{\pi })\right)\right)+\frac{v_{\delta }}{1+\frac{p_{a}}{k_{\delta }}}Hill(c_{a}^{2},K_{PLC\delta })\\ +v_{3K}Hill(c_{a}^{4},K_{D})Hill(p_{a},K_{3})-r_{5p_{a}}p_{a}\\ \frac{dh_{a}}{dt}=\alpha_{h_{a}}(1-h_{a})-\beta_{h_{a}}h_{a} \end{array}$$ (A-9)

The first term of above equation denotes agonist-dependent (glutamate) *IP*_3_ production; the second term shows agonist-independent *IP*_3_ production which is due to *PLCδ* signaling and modulated by *Ca*^2+^, the third term shows *IP*_3_ degradation rate by *IP*_3_ − 3*k* which is *Ca*^2+^ dependent and the last term incorporates degradation of *IP*_3_ by *IP* − 5*P*. $\alpha_{h_{a}}$ and $\beta_{h_{a}}$ denote opening and closing rate of *h*_*a*_ respectively. Other parameters in mentioned equation can be determined using equations:

$$m_{\infty ,a}=Hill(p_{a},d_{1}),n_{\infty ,a}=Hill(c_{a},d_{5}),Hill(x^{n},K)=\frac{x^{n}}{x^{n}+K^{n}},\alpha_{h_{a}}=a_{2}d_{2}\frac{p_{a}+d_{1}}{p_{a}+d_{3}},\beta_{h_{a}}=a_{2}c_{a}$$ (A-10)

Further information about the parameters and their values can be found in De pitta’s paper [46].

#### Postsynaptic neuron

AMPAR current governs by the following equations:

$$I_{AMPA}=f_{AMPA}(g_{AMPA},m_{AMPA},{\text{V}}_{post})=g_{AMPA}m_{AMPA}({\text{V}}_{post}-{\text{V}}_{AMPA})$$ (A-11)

Where V_*AMPA*_ is reversal potential of the receptor, V_*post*_ denotes membrane potential, *m*_*AMPA*_ represents gating variable of AMPAR. Since we assume that both presynaptic and astrocytic glutamate have effects on postsynaptic neuron, so gating variable of AMPAR can be described by:

$$\frac{dm_{AMPA}}{dt}=\alpha_{AMPA}(a_{1}{\text{g}}_{pre}+a_{2}{\text{g}}_{ast})(1-{\text{m}}_{AMPA})-\beta_{AMPA}m_{AMPA}$$ (A-12)

Here *α*_*AMPA*_ and *β*_*AMPA*_ are opening and closing rate of the receptor, *g*_*pre*_ and *g*_*ast*_ denote glutamate concentration released by presynaptic neuron and astrocyte respectively, *a*_1_ = 0.42 and *a*_2_ = 0.01 are constants.

In Eq (A-11) AMPAR channel’s conductance (*g*_*AMPA*_) can be varied due to postsynaptic CaMKII. This process modeled using modified equations [50, 51]:

$$EP1(\text{Ph}.\text{CaMKII})=EP2(\text{Ph}.CaMKII)=1+\frac{30{\left(\text{Ph}.\text{CaMKII}\right)}^{2}}{\left(1+{\left(\text{Ph}.\text{CaMKII}\right)}^{2}\right)},EK1(\text{Ph}.\text{CaMKII})=EK2(\text{Ph}.\text{CaMKII})=1+\frac{100{\left(\text{Ph}.\text{CaMKII}\right)}^{2}}{\left(8^{2}+{\left(\text{Ph}.\text{CaMKII}\right)}^{2}\right)}$$ (A-13)

Here, the activity of protein kinase and protein phosphatase assumed to be a hill function of CaMKII and have been shown by *EK* and *EP* respectively [51].

$$\begin{array}{l} A=\frac{A_{T}.EP1.EP2}{\left(\text{EK}2+\text{EP}2)(\text{EK}1+\text{EP}1\right)},A_{P_{1}}=\frac{A_{T}.EK1.EP2}{\left(\text{EK}2+\text{EP2})(\text{EK}1+\text{EP}1\right)},A^{P_{2}}=\frac{A_{T}.EP1.EK2}{\left(\text{EK}2+\text{EP}2)(\text{EK}1+\text{EP}1\right)},A_{P_{1}}^{P_{2}}=\frac{A_{T}.EK1.EK2}{\left(\text{EK}2+\text{EP}2)(\text{EK}1+\text{EP}1\right)}\\ A_{T}=A+A_{p_{1}}+A^{P_{2}}+A_{P_{1}}^{P_{2}} \end{array}$$ (A-14)

Here, *A*, $A_{p_{1}}$, $A^{P_{2}}$ and $A_{P_{1}}^{P_{2}}$ are reaction substrates and products. The conductance of AMPAR can be determined using the below equation:

$$g_{AMPA}=f_{g_{AMPA}}(\text{CaMKII})=A+2(A_{P_{1}}+A^{P_{2}})+4A_{P_{1}}^{P_{2}}$$ (A-15)

Further information about AMPAR phosphorylation can be found in Castellani et all’s papers [50, 51].

Furthermore, in the proposed model, *I*_*NMDA*_ modeled using Moradi et all’s formulation with a slight modification:

$$I_{NMDA}=f_{NMDA}(g_{NMDA},m_{NMDA},Mg,{\text{V}}_{post})=g_{NMDA}m_{NMDA}Mg({\text{V}}_{post}-{\text{V}}_{NMDA})$$ (A-16)

Here, Mg denotes *Mg*^2+^ blocking and is governed by:

Mg=fMg(Morph,Vpost)=1/(1+[Mg2+]0(k0+15.581+(0.1Morph)1.2)−1exp(−z(δ+0.11+(0.1Morph)1.2)FVpostR−1T−1)) (A-17)

Moreover, NMDAR gating variable is governed by:

$$\frac{dm_{NMDA}}{dt}=f_{m_{NMDA}}(\alpha_{NMDA},\beta_{NMDA},g_{pre},g_{Astro})=\alpha_{NMDA}\left(k_{1}g_{pre}+k_{2}g_{Astro}\right)\left(1-m_{NMDA}\right)-\beta_{NMDA}\;m_{NMDA}$$ (A-18)

Here V_*NMDA*_ is the reversal potential of NMDAR, *α*_*NMDA*_ and *β*_*NMDA*_ are opening and closing rate of receptor respectively, *g*_*pre*_ and *g*_*Astro*_ are glutamate concentrations released by presynaptic pyramidal neuron and astrocyte respectively. *g*_*NMDA*_ is NMDA channels conductance which is described by:

gNMDA=fgNMDA(Morph,gVD)=(gVI+0.151+(0.1Morph)1.2)+gVD (A-19)

Here *g*_*VI*_ is the channel conductance independent of potential and *g*_*VD*_ is the channel conductance dependent to the potential which can be written by:

$$\begin{array}{l} \frac{dg_{VD}}{dt}=(g_{VD,\infty }-g_{VD})/\tau_{g}\\ g_{VD,\infty }=k({\text{V}}_{post}-{\text{V}}_{0}) \end{array}$$ (A-20)

Where *g*_*VD*,∞_ is the final value of *g*_*VD*_ with time constant of *τ*_*g*_ and has a linear relation with membrane potential with constant of k. Other parameters and values listed in S1 Table.

Postsynaptic calcium oscillations which introduced in Eq (28) can be determined by [22]:

$$\frac{dc_{post}}{dt}=f_{Ca_{Post}}({\text{I}}_{AMPA},{\text{I}}_{NMDA},{\text{I}}_{CaL},{\text{S}}_{Pump})=\frac{f\left(c_{post}\right)}{1+\theta },f\left(c_{post}\right)=-\frac{\left(\eta I_{AMPA}+\gamma I_{NMDA}+I_{L}\right)}{z_{ca}FV_{spine}}-s_{pump},s_{pump}=k_{s}\left(c_{post}-c_{post}^{rest}\right),\theta =\frac{b_{t}K_{endo}}{{\left(K_{endo}+c_{post}\right)}^{2}},I_{L}=g_{L}B(\text{N},{\text{P}}_{open})({\text{V}}_{post}-{\text{V}}_{L})$$ (A-21)

Here, *c*_*post*_ denotes postsynaptic calcium concentration which depends on AMPAR current, and is denoted by *I*_*AMPA*_, NMDA current is shown by *I*_*NMDA*_, voltage-gated calcium channels activation is described by *I*_*L*_ and pumped calcium defined by *s*_*pump*_. Here, *η* = 0.012 and *γ* = 0.06 are the constants for AMPAR and NMDAR mediated calcium ions, *z*_*ca*_ = 2 denotes calcium valence, *F* = 96487*C* / *mole* is the faraday’s constant, *V*_*spine*_ = 0.9048*um*^3^ is the volume for dendrite spin, *k*_*s*_ = 100 / *s* maximum efflux rate of calcium pump, $c_{post}^{rest}=100nM$ rest value for postsynaptic calcium concentration, *b*_*t*_ = 200*uM* denotes total endogenous buffer concentration and *K*_*endo*_ = 10*uM* shows endogenous buffer calcium affinity. Also, *g*_*L*_ = 15*ps* is the conductance of calcium channel current, *B*(N, P_*open*_) is a random variable with a binomial distribution that shows the number of opened channels and *V*_*L*_ = 27.4*mv* describes the reversal potential.

Postsynaptic *Ca*^2+^ concentration variation may lead to CaMKII phosphorylation which is governed by equations [52]:

dP0dt=−v1+v3P1,dP1dt=v1−v3P1−v2P1+2v3P2,dP2dt=v2P1−2v3P2−1.8v2P2+3v3P3dP3dt=1.8v2P2−3v3P3−2.3v2P3+4v3P4,dP4dt=2.3v2P3−4v3P4−2.7v2P4+5v3P5dP5dt=2.7v2P4−5v3P5−2.8v2P5+6v3P6,dP6dt=2.8v2P5−6v3P6−2.7v2P6+7v3P7dP7dt=2.7v2P6−7v3P7−2.3v2P7+8v3P8,dP8dt=2.3v2P7−8v3P8−1.8v2P8+9v3P9dP9dt=1.8v2P8−9v3P9−v2P9+10v3P10,dP10dt=v2P9−10v3P10depdt=−k3Iep+k4(ep0−ep),dIdt=−k3Iep+k4(ep0−ep)+vPKAI0−vCaN([Ca2+]/KH2)3I1+([Ca2+]/KH2)3 (A-22)

Here, *P*_*i*_ denotes the concentration of the i-fold phosphorylated CaMKII, *e*_*p*_ shows the PP1 concentration which not bounded to l1P and can demonstrate active protein phosphatase, the total concentration of PP1 is denoted by *e*_*p*0_ = 0.1*μM*, free l1P is shown by *I*, free l1 concentration is demonstrated by *I*_0_ = 0.1*μM*. Association and dissociation rate constant of PP1-l1P complex are denoted by *k*_3_ = 1 / *μMs* and *k*_4_ = 10^−3^ / *s* respectively. ν_*CaN*_ = 2 / *s* is the rate of l1P dephosphorylation due to CaN (calcineurin), v_*PKA*_ = 0.45*μM* / *s* is the phosphorylation rate of l1 due to PKA, *K*_*H*2_ = 0.7*μM* is the calcium activation Hill constant of CaN.

The rate of phosphorylation (*ν*_1_), auto-phosphorylation (*ν*_2_) and dephosphorylation (*ν*_3_) can describe by:

$$v_{1}=\frac{10k_{1}{\left([{\text{Ca}}^{2+}]/{\text{K}}_{H1}\right)}^{8}P_{0}}{{\left(1+{\left([{\text{Ca}}^{2+}]/{\text{K}}_{H1}\right)}^{4}\right)}^{2}},v_{2}=\frac{k_{1}{\left([{\text{Ca}}^{2+}]/{\text{K}}_{H1}\right)}^{4}}{1+{\left([{\text{Ca}}^{2+}]/{\text{K}}_{H1}\right)}^{4}},v_{3}=\frac{k_{2}e_{p}}{K_{M}+\sum_{1}^{10}iP_{i}}$$ (A-23)

Here, *k*_1_ = 0.5 / *s* is the l1 dependent regulation rate of PP1 and *K*_*H*1_ = 4*μM* is the hill constant of CaMKII for calcium activation. *K*_*M*_ = 20*μM* And *k*_2_ = 10 / *s* are the Michaelis and catalytic constants respectively. Then, phosphorylated CaMKII can be described by:

$$Ph.CaMKII=f_{CaMKII}({\text{c}}_{Post})=\sum_{i=1}^{10}P_{i}$$ (A-24)

## Supporting information

S1 Table Postsynaptic neuron parameters. (DOCX)
