# Supplementary material for: Computational modeling of opioid-induced synaptic plasticity in hippocampus
Source: PLoS One. 2018 Mar 7;13(3):e0193410. doi: 10.1371/journal.pone.0193410 (PMC5841814; doi:10.1371/journal.pone.0193410)
Supplement: S1 Table — (DOCX) [file pone.0193410.s001.docx]

**Supplemental 1 Table. Postsynaptic neuron parameters.**

| variable | Description | Value (units) | source |
| --- | --- | --- | --- |
| *R_m_* | Actual resistance of the spine head | $0.79*{10}^{5} M\Omega$ | [48] |
| $V_{post}^{rest}$ | Postsynaptic resting membrane potential | 70- mV | [48] |
| $\tau_{post}$ | Postsynaptic membrane time constant | 50 ms | [48] |
| $g_{AMPA}$ | AMPAR conductance before NO production | 0.35 nS | [49] |
| $V_{AMPA}$ | AMPAR reversal potential | 0 mV | [49] |
| $\alpha_{AMPA}$ | AMPAR forward rate constant | 1.1 $\mu Ms^{-1}$ | [49] |
| $\beta_{AMPA}$ | AMPAR backward rate constant | $190 s^{-1}$ | [49] |
| $V_{NMDA}$ | NMDAR reversal potential | 0 mV | [49] |
| $F$ | Faraday’s constant | $96487 C {mole}^{-1}$ | [53] |
| $R$ | Real gas constant | 8.314 J/K | [53] |
| $T$ | Absolute temperature | 293.15 K | [53] |
| $z$ | Valence of $\mathrm{Mg}^{2+}$ | 2 | [53] |
| $\delta$ | Relative electrical distance of the binding site of $\mathrm{Mg}^{2+}$ from the outside of the membrane | 0.8 | [53] |
| $K_{0}$ | $IC_{50}$at 0 mV | 4.1 mM | [53] |
| $[\mathrm{Mg}^{2+}]_{0}$ | Concentration of $\mathrm{Mg}^{2+}$in the extracellular fluid | 1 mM | [53] |
| $k_{1\text{ }}$ | Constant value | 0.28 | Proposed model |
| $k_{2}$ | Constant value | 0.05 | Proposed model |
| $\alpha_{NMDA}$ | NMDAR forward rate constant | $7.2*10^{4}$ $M^{-1}s^{-1}$  | [49] |
| $\beta_{NMDA}$ | NMDAR backward rate constant | 6.6 $s^{-1}$ | [49] |
| $k$ | Steepness of speed of Voltage-dependent gating of channel | 0.007 | [53] |
| $V_{0}$ | The $V_{m}$at which $g_{VD,\infty}$ is equal to zero | 100- mV | [53] |
| $\tau_{g}$ | Time constant which determines the speed of Voltage-dependent gating of channel | 0.05 ms | [53] |
